# Retrospective View of North American Potato (*Solanum tuberosum* L.) Breeding in the 20^th^ and 21^st^ Centuries

**DOI:** 10.1534/g3.113.005595

**Published:** 2013-06-01

**Authors:** Candice N. Hirsch, Cory D. Hirsch, Kimberly Felcher, Joseph Coombs, Dan Zarka, Allen Van Deynze, Walter De Jong, Richard E. Veilleux, Shelley Jansky, Paul Bethke, David S. Douches, C. Robin Buell

**Affiliations:** * Department of Plant Biology, Michigan State University, East Lansing, Michigan 48824; † Department of Plant, Soil and Microbial Sciences, Michigan State University, East Lansing, Michigan 48824; ‡ Seed Biotechnology Center, University of California, Davis, California 95616; § Department of Plant Breeding & Genetics, Cornell University, Ithaca, New York 14853; ** Department of Horticulture, Virginia Polytechnic Institute and State University, Blacksburg, Virginia 24061; †† Department of Horticulture, University of Wisconsin, Madison, Wisconsin, 53706; ‡‡ United States Department of Agriculture-Agricultural Research Service, Vegetable Crops Research Unit, Madison, Wisconsin, 53706

**Keywords:** *Solanum tuberosum*, phenotypic diversity, genotypic diversity, genomics

## Abstract

Cultivated potato (*Solanum tuberosum* L.), a vegetatively propagated autotetraploid, has been bred for distinct market classes, including fresh market, pigmented, and processing varieties. Breeding efforts have relied on phenotypic selection of populations developed from intra- and intermarket class crosses and introgressions of wild and cultivated *Solanum* relatives. To retrospectively explore the effects of potato breeding at the genome level, we used 8303 single-nucleotide polymorphism markers to genotype a 250-line diversity panel composed of wild species, genetic stocks, and cultivated potato lines with release dates ranging from 1857 to 2011. Population structure analysis revealed four subpopulations within the panel, with cultivated potato lines grouping together and separate from wild species and genetic stocks. With pairwise kinship estimates clear separation between potato market classes was observed. Modern breeding efforts have scarcely changed the percentage of heterozygous loci or the frequency of homozygous, single-dose, and duplex loci on a genome level, despite concerted efforts by breeders. In contrast, clear selection in less than 50 years of breeding was observed for alleles in biosynthetic pathways important for market class-specific traits such as pigmentation and carbohydrate composition. Although improvement and diversification for distinct market classes was observed through whole-genome analysis of historic and current potato lines, an increased rate of gain from selection will be required to meet growing global food demands and challenges due to climate change. Understanding the genetic basis of diversification and trait improvement will allow for more rapid genome-guided improvement of potato in future breeding efforts.

Cultivated potato, *Solanum tuberosum* L., is grown for its starchy tuber, a below-ground storage organ that provides a natural means of asexual reproduction. A collection of related taxonomic groups exists within *S. tuberosum*. Several of these groups, Andigena, Phureja, and Stenotomum, were domesticated in the Andean region of South America and many will not form tubers under long-day conditions typical of summers in North America and Europe. In contrast, *S. tuberosum* Group Tuberosum is widely grown throughout the world and has been adapted to form tubers under long-day conditions. Nearly all potato cultivars are heterozygous autotetraploids (2*n* = 4*x* = 48) that are vegetatively propagated for germplasm maintenance and agricultural production. Flowering and seed development occurs to varying degrees in most cultivars, but true seeds are only of interest to potato breeders, as they generally give rise to progeny that exhibit inbreeding depression in the form of lost agronomic performance. Wild species related to potato, including *Solanum chacoense* and *Solanum tarijense*, are typically diploid (2*n* = 2*x* = 24) and reproduce sexually through outcrossing and asexually through tubers.

Cultivated potato in North America has a narrow genetic base. The short-day photoperiod adaptation of germplasm from South America, the potato’s center of origin, initially limited its direct use in varietal breeding. Epidemics of late blight in the mid-1800s also reduced the number of cultivated lines that could be used for effective breeding. A decrease in sexual fertility of vegetatively propagated cultivars that might be used as parents, combined with low vigor due to virus accumulation over years of tuber propagation, further restricted the germplasm used in breeding efforts. In addition, severe inbreeding depression has been observed when cultivated potato is self-pollinated, a phenomenon attributed to the retention of deleterious and dysfunctional alleles (Mullin and Lauer 1966). This hypothesis is consistent with genome analyses that revealed a higher frequency of frameshift mutations in a vigorous heterozygous diploid line compared with a low-vigor, homozygous doubled monoploid line (Potato Genome Sequencing Consortium 2011).

Contemporary breeding efforts for cultivated potato rely on phenotypic rather than genotypic selection of populations developed from intra- and intermarket class crosses, introgressions of wild and cultivated *Solanum* relatives, and ploidy manipulations. Due to sterility and inbreeding depression, superior phenotypes are selected in the F_1_ generation, and evaluated as clones over 10−15 years. Thus, whatever genetic recombination occurred during meiosis in the parental lines is fixed with no opportunity for additional genetic recombination or major genetic changes during subsequent years of evaluation.

It has been proposed that maximizing heterosis for yield in potato may be achieved by maximizing heterozygosity and associated intra- and interlocus interactions (Mendoza and Haynes 1974). Several studies support this hypothesis (Mendiburu and Peloquin 1971; Mok and Peloquin 1975; De Jong and Tai 1977; Buso *et al.* 1999), and efforts have been made to increase heterozygosity by introgressing allelic diversity from other *Solanum* species into cultivated potato (Haynes 1980; Hermundstad and Peloquin 1987; Plaisted and Hoopes 1989; Bradshaw and Ramsay 2005; Jansky and Peloquin 2006). Heterozygosity itself, however, is not necessarily highly desirable if its source introduces undesirable traits (Bani-Aameur *et al.* 1991).

Despite the narrow genetic base, cultivated potato is bred for a wide range of market classes. Before 1950, there were only round white- and red-skinned table market types. After 1950, the potato market classes expanded to meet the needs of the developing French fry processing industry, which requires elongated tubers with long dormancy; and the stringent requirements of the chip-processing industry, including high starch content and low tuber reducing sugar content at harvest and after storage. Both chip and fry processing cultivars also have been bred for increased cold sweetening resistance, which limits the accumulation of reducing sugars in tubers when they are stored at temperatures below approximately 10° for extended periods (Sowokinos *et al.* 1987). The market types also expanded to add table russets, used for home and restaurant baking, and pigmented specialty varieties that are defined by their shape as well as their flesh and skin coloration. Yellow-fleshed cultivars entered the U.S. market in the late 1980s, followed by red, blue, and other pigmented specialty types. Populating these diverse market classes was possible due to ample phenotypic diversity within the *S. tuberosum* Groups, which can be readily crossed with commercial cultivars.

Potato is the third most important food crop worldwide, behind wheat and rice (http://faostat.fao.org/site/339/default.aspx) in large part because of its adaptability, yield potential, and nutritional advantages relative to other crops. New potato cultivars with increased yield and improved performance under biotic and abiotic stress will be needed to keep up with increasing global demand for food and the effects of climate change. With the release of the potato genome reference sequence (Potato Genome Sequencing Consortium 2011), and the development of genomic resources for potato (Hamilton *et al.* 2011; Felcher *et al.* 2012), it is now possible to systematically assess the genetic diversity in cultivated potato to identify genomic regions, genes, and alleles contributing to agronomic traits of interest and to develop molecular markers for efficient marker-assisted selection. As part of the Solanaceae Coordinated Agricultural Project (http://solcap.msu.edu), we assembled a diverse panel of 250 potato lines that included historical and contemporary cultivars in all market classes, advanced breeding lines, wild species, and genetic stocks from breeding programs around the world. The panel was genotyped using the SolCAP 8303 SNP Infinium array (Felcher *et al.* 2012), and the single-nucleotide polymorphism (SNP) data were used to assess genetic structure and divergence, the extent of heterozygosity within wild and cultivated potato, and the effects that breeding for specific market classes had on allelic composition in the potato genome.

## Materials and Methods

### Germplasm

The germplasm used for this study included 250 diverse potato lines (Supporting Information, Table S1). Market class designations in the panel were determined by current or past market use of named varieties and by breeder classifications for advanced breeding lines [Chip Processing (69), French Fry Processing (34), Pigmented (32), Round White Table (38), Table Russet (13) and Yellow (27)]. Some lines could be classified into two market classes; however, they were assigned to only a single market class. For yellow-fleshed lines, the Yellow market class was prioritized, followed by the Pigmented market class. For dual-purpose russet lines (both processing and table), the processing market (French Fry Processing) was the primary class, and Round White Table was prioritized over Chip Processing (Figure S1, Table S1). Twenty-five genetic stocks and 12 wild species also were included in the panel. The historical varieties were obtained from the NRSP-6 potato gene bank as tissue culture plants and the Michigan State University Potato Breeding and Genetics Program propagated tubers for the field evaluation. For all other lines, North American breeding programs supplied tubers for the field evaluations. The wild species and cultivated potato lines used in this study are publically available as tissue culture plants through author D.S.D. (Michigan State University) or NRSP-6, Sturgeon Bay, WI. The advanced breeding lines can be obtained directly from the breeding programs as noted in Table S1.

### SNP genotyping

The 250 lines in the diversity panel were genotyped with the Infinium 8303 Potato Array (Felcher *et al.* 2012), which provides genome-wide representation of SNPs present in cultivated potato germplasm. DNA was extracted from young leaf tissue from individual tissue culture plants, greenhouse grown plants, or field plants using the QIAGEN DNeasy Plant Mini Kit (QIAGEN, Germantown, MD), quantified using the Quant-iT PicoGreen dsDNA Assay Kit (Invitrogen, San Diego, CA), and adjusted to a concentration of 50 ng·µL^−1^. The diversity panel was assayed using an Illumina iScan Reader (Illumina, San Diego, CA) and the Infinium 8303 Potato Array according to the manufacturer’s suggested protocol. Data were analyzed in the Illumina GenomeStudio software (Illumina, San Diego, CA).

A custom cluster file was generated within GenomeStudio, which assumes a diploid model and three marker classes for each SNP (AA, AB, and BB). An additional custom file was generated for a tetraploid model with five marker classes for each SNP accounting for dosage (AAAA, AAAB, AABB, ABBB, and BBBB) based on theta values. A set of 354 diverse lines and three mapping populations [two F_1_ tetraploid mapping populations (Premier Russet × Rio Grande Russet and MSG227 × Jacqueline Lee) and one F_1_ diploid mapping population (DM1-3 516 R44 × 84SD22) (Felcher *et al.* 2012)] were included in the determination of cluster positions for the diploid and tetraploid models to allow for more accurate placement of cluster positions by use of parental line genotype scores and expected segregation ratios in the F_1_ progeny. Boundaries of cluster positions for each marker class were determined and a custom Perl script was used to convert a matrix of theta values exported from GenomeStudio into actual genotype scores (Figure S2). Marker class theta boundaries could only be determined for the marker classes that were present in the set of diverse lines and segregating populations. The diploid model cluster file (http://solcap.msu.edu) and the positions of the tetraploid model boundaries (Table S2) are provided.

Using the aforementioned diploid and dosage cluster files, we determined genotype calls for our 250-line diversity panel. Of these, 217 were independent DNA replicates of lines used to generate the cluster files. The average number of loci with contradicting calls within these replicates was only 0.2% with a maximum of 0.6% in the diploid model. In contrast, for the dosage model, the average difference was 1.7% with a maximum of 5.4%. These results suggest that the dosage model may be affected by thermodynamic efficiency and fluorescence consistency. The 160 SNPs in the glycoalkaloid, carotenoid, and carbohydrate pathways and the random SNPs described in this study were manually annotated using the 250 lines in the panel.

While determining the cluster positions, we evaluated the quality of each SNP, and SNPs with low-signal intensity, loose clustering, or other assay failures were filtered out. After removing low-quality SNPs, there were 7666 SNPs in the diploid three cluster model. For the tetraploid model, there were 2645 SNPs with boundaries for all five clusters, 858 SNPs with boundaries for four of the five clusters, 945 SNPs with boundaries for three of the clusters, and 583 SNPs with boundaries for two of the clusters. The remaining SNPs were removed due to low quality or inability to clearly distinguish between the three heterozygous marker classes (AAAB, AABB, and ABBB). To determine whether SNPs mapped to multiple places in the genome, 50 bp of context sequence on both sides of the SNP were aligned to the version 2.1.11 pseudomolecules (http://solanaceae.plantbiology.msu.edu/pgsc_download.shtml) with the est2genome model within exonerate version 2.2.0 (Slater and Birney 2005), using a minimum intron size of 10 bp and a maximum intron size of 15,000 bp. Alignments were required to have 95% sequence identity, 95% coverage, and no insertions or deletions. SNPs that mapped to two or more places in the genome were removed from future analysis (733 SNPs). Finally, a SNP was removed if there was greater than 20% missing data. After filtering, 6373 SNPs remained using the diploid method (Table S3) and 3763 SNPs using the dosage method (Table S4).

### Genotypic data analysis

Population structure was determined with the STRUCTURE software (Pritchard *et al.* 2000) using an admixture model. For each dataset three replicates were performed for each value of *K* (number of populations) from 1 to 10 with a burn-in time and number of Markov Chain Monte Carlo replicates after burn-in set to 50,000. The optimal value of *K* was determined as the *K* with the maximum likelihood of the observed genotypes given the number of subpopulations in the model, and a single replicate from that *K* was used to assign the probability that a line belonged to each substructure group.

Pairwise measures of genetic relatedness were calculated as Roger’s distances (Rogers 1972) using the dosage dataset. PowerMarker version 3.25 (Liu and Muse 2005) was used to construct the unweighted pair group method with arithmetic mean (UPGMA) based tree using the Roger’s distance dissimilarity matrix. TreeView version 1.6.6 was used to generate the tree image (Page 1996). Market class designations based on pedigree and phenotypic observations (Table S1) were used to determine branch coloring within the tree.

Enrichment tests of market class selected alleles and genotypes were done using a χ^2^ test. For SNPs in carotenoid biosynthetic pathway genes, the observed frequencies were determined from the Yellow lines, and the expected frequencies were determined from all other cultivated potato market classes (Chip Processing, French Fry Processing, Pigmented, Round White Table, and Table Russet). For SNPs in carbohydrate-associated genes, the observed frequencies for the Chip Processing tests were determined from the Chip Processing lines and the expected frequencies from all other cultivated potato market classes (French Fry Processing, Pigmented, Round White Table, Table Russet, and Yellow), and the observed frequencies for the French Fry Processing tests were determined from the French Fry Processing lines and the expected frequencies from all other cultivated non-processing lines (Pigmented, Round White Table, Table Russet, and Yellow). Allele and genotype frequencies were calculated using the dosage model in the tetraploid cultivated potato lines. Within an individual, the allele frequency values can be 0, 0.25, 0.5, 0.75, or 1 for A and B. The allele frequency test used one degree of freedom, and degrees of freedom used in genotype frequency test varied by how many marker classes were present, with a maximum of four degrees of freedom. For SNPs in glycoalkaloid pathway candidate genes, the observed frequencies were determined from all cultivated potato (Chip Processing, French Fry Processing, Pigmented, Round White Table, Table Russet, and Yellow) and the expected frequencies were observed from the wild species. Allele and genotype frequencies were calculated using the diploid model and individual allele frequency values could be 0, 0.5, or 1 for A and B. The allele frequency test used one degree of freedom, and degrees of freedom used in the genotype frequency test varied by how many marker classes were present, with a maximum of two degrees of freedom. For all comparisons, random SNPs throughout the genome were selected to test if observations in the selected SNPs were observed in a random set of SNPs. For both the carotenoid biosynthetic pathway SNPs and carbohydrate-associated SNPs, the frequency of significant SNPs was higher in the tested SNPs compared to the random SNPs (Table S5 and Table S6). For the glycoalkaloid SNPs, the frequency of significant SNPs was the same or greater in the random SNPs compared to the tested SNPs (Table S7).

### Field evaluation

Field trials were grown during the summer of 2010 at the Hancock Agricultural Research Station in Hancock, WI, and Cornell University in Ithaca, NY. The soil type at the WI location is Plainfield loamy sand soil and at the NY location is Chenango gravelly loam. Three replicates of the experiment were grown. Two replicates were grown in a randomized complete block design at the Wisconsin location and one randomized replicate was grown at the New York location. Plots consisted of 10 plants and were grown with best management practices for each location. The WI location was planted on April 28, 2010, and harvested on September 10, 2010, and the NY location was planted on April 29, 2010, and harvest on September 8, 2010.

Tubers were stored at 10°F and processed 21 d after harvest. For tuber sucrose concentration and tuber glucose concentration, eight unblemished tubers from each plot were cored with a #4 cork borer and a 1 cm section was taken from each core avoiding both the periderm and the pith. Samples (10 core sections/plot) were placed in 15-mL conical tubes, immediately frozen in liquid nitrogen and lyophilized. Biochemical sample preparation and high-performance liquid chromatography analysis was conducted as previously described (Bethke *et al.* 2009), except that the column was cooled to 20° to minimize acid hydrolysis of sucrose. Sugar content was reported as mg·g^−1^ dry weight. For Snack Food Association (SFA) chip color analysis, cored tubers were cut in half from the basal to the apical end and two slices (1.8 mm thick) were cut with a mandolin slicer. Potato slices were fried in heavy duty peanut oil (Sysco, Houston, TX) at 185°F for approximately 2.5 min or until bubbling stopped. Chips were visually scored for SFA chip color using the SFA scale in, which one is the lightest and five is the darkest (www.sfa.org/products; Figure S3). For tuber shape, 10 tubers were scored using a one to five scale where one is compressed and five is long (Figure S4).

### Phenotypic data analysis

Quality of the raw phenotypic data was assessed using PROC UNIVARIATE in SAS (SAS Institute 2003). Phenotypic analysis was performed using PROC GLM in SAS to partition variation due to replication and line for each phenotypic trait (Table S8) and to generate least square means for each line across replications (Table S9). All sources of variation were considered random, and all statistical tests were conducted at a 5% level of significance. Normality of the residuals for each trait was assessed with PROC UNIVARIATE in SAS. Spearman rank correlations were determined using PROC CORR (SAS Institute 2003) for traits with a significant replication effect to determine whether the replicates could be combined. Scatter plots of phenotypic data were generated using R version 2.13.2 (Ihaka and Gentleman 1996).

## Results and Discussion

### Germplasm description

**Figure 1:**
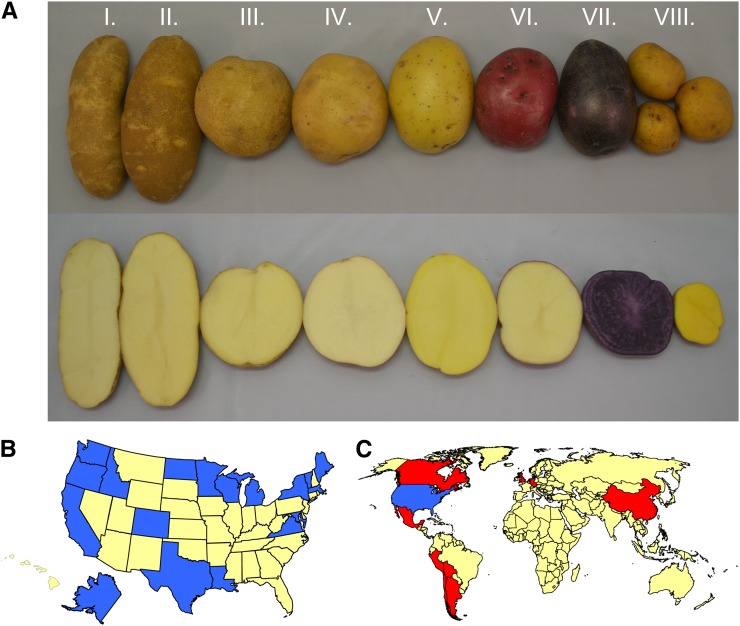
Phenotypic and geographic diversity represented within the diversity panel. (A) Representative lines from each of the market classes (I. French Fry Processing, II. Table Russet, III. Chip Processing, IV. Round White Table, V. Yellow, VI. Pigmented (red skin with white flesh), VII. Pigmented (purple skin with purple flesh), and VIII. Genetic Stock). Geographic distribution within the United States (B) and worldwide (C) are based on breeding program from which the line was released and collection location. Blue shading indicates U.S. states that are represented in the diversity panel, red shading indicates countries that are represented in the diversity panel, and yellow shading indicates states or countries that are not represented in the diversity panel.

The diversity panel evaluated in this study represents a broad range of genotypic diversity, phenotypic diversity (Figure 1A), geographic origin (Figure 1, B and C), ploidy levels (1*x*, 2*x*, and 4*x*), wild species used for introgressions, release dates, and market classes (Table S1). Cultivated lines represent breeding efforts prior to 1900 (five varieties), breeding efforts before the advent of the frozen French fry processing industry (1900−1950; 10 lines), and breeding efforts focused on specific market classes subsequent to 1950 (119 released lines) including Chip Processing (36 lines), French Fry Processing (21 lines), Table Russet (nine lines), yellow-fleshed varieties (hereafter referred to as the “Yellow” market class) released since the introduction of Yukon Gold in 1981 (16 lines), round white table varieties (16 lines) and Pigmented specialty types that have been grown commercially since 1990 (15 lines). In some instances, lines were placed into multiple market classes [*e.g.*, Peter Wilcox – yellow-fleshed (Yellow) and purple skinned (Pigmented)]. In addition, 12 wild *Solanum* species and primitive cultivars, hereafter referred to collectively as “wild species,” and 25 genetic stock lines, only a few generations removed from the wild species, were included (Table S1). Lines were selected from 13 public breeding programs within the United States (Figure 1B), and from prominent breeding programs in 10 other countries in North America, South America, Europe, and Asia (Figure 1C) to provide a broader set of germplasm. Although the totality of phenotypic and genotypic diversity within tuber-bearing *Solanum* species cannot be represented within a single diversity panel, the 250 lines in this panel maximize diversity of the metrics described previously.

The relatively large number of processing lines in this study provided the opportunity to examine phenotypic and genotypic characteristics such as chip color (Figure S3) and tuber shape (Figure S4) within the processing market classes. The stringent market requirements for Chip Processing varieties (light chip color, low defects, high starch content, and resistance to cold-sweetening) coupled with dedicated breeding for a single product created an opportunity for substantial trait improvement compared to the initial cultivars bred before 1950. In contrast, the frozen French fry processing industry was developed around Russet Burbank and market acceptance of new cultivars is based upon maintaining the processing and culinary features of ‘Russet Burbank.’ As a consequence, varietal improvement has focused primarily on increased resistance to disease and abiotic stress and increased yield of marketable tubers per hectare planted.

### Genetic structure and divergence between market classes

**Figure 2:**
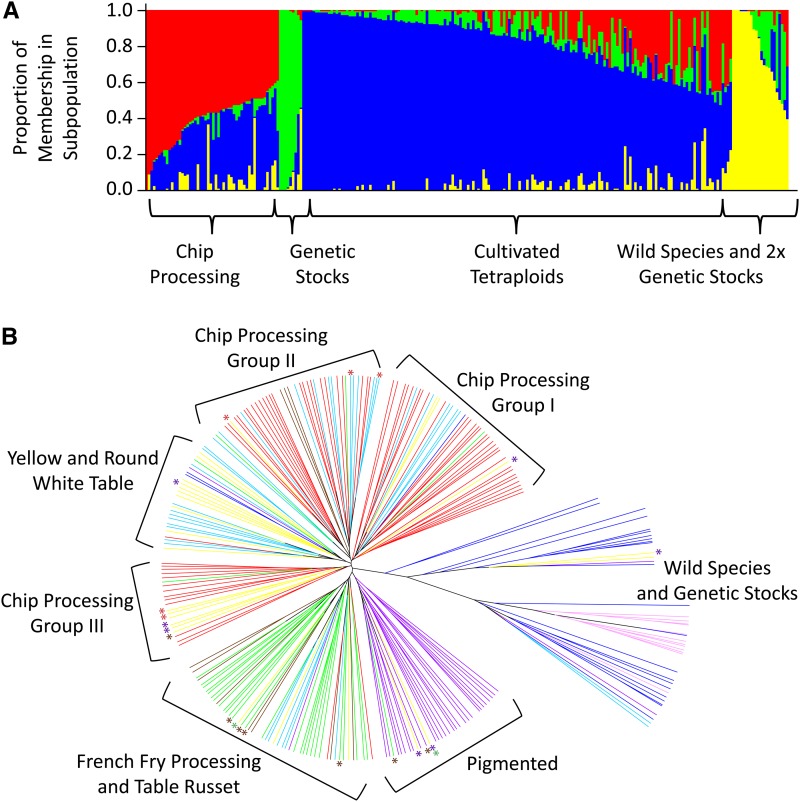
Genetic structure and divergence between market classes. (A) Graphical display of population substructure for 250 lines based on 6373 SNP markers using the diploid genotype calls with *K* = 4 populations. Population substructure was determined using STRUCTURE (Pritchard *et al.* 2000). Each vertical bar represents one individual in the population and each individual is partitioned into the four possible subpopulations based on the percentage of membership in each subpopulation. Subpopulation 1: red (30/41 with majority membership Chip Processing); subpopulation 2: green (7/8 with majority membership Genetic Stock); subpopulation 3: blue (157/160 with majority membership cultivated potato); subpopulation 4: yellow (19/19 with majority membership wild species or genetic stock)). (B) Unweighted pair group method with arithmetic mean (UPGMA) tree of 250 potato lines based on 3763 SNP markers with dosage genotype calls. Color-coding is based on predominant market class designation and asterisks indicate lines that can be classified into more than one market class (red: Chip Processing; dark blue: Genetic Stock; purple: Pigmented; green: French Fry Processing; light blue: Round White Table; pink: wild species; brown: Table Russet; yellow: Yellow).

Population structure within the diversity panel was examined using STRUCTURE (Pritchard *et al.* 2000), with *K* (number of subpopulations) ranging from zero to 10 using the diploid genotyping model (AA, AB, BB; 6373 SNP markers). The *K* value with the maximum likelihood was four (Figure 2A, Figure S5). Lines were assigned to a subpopulation if they had at least 50% membership within that group. Most of the cultivated, tetraploid lines grouped together (157/160 lines in subpopulation 3, blue; Figure 2A, Table S1). The genetic stocks and wild species were distinctly different from tetraploid cultivated potato (7/8 lines in subpopulation 2, green; 19/19 lines in subpopulation 4, yellow; Figure 2A, Table S1). Chip Processing lines showed some outgrouping, as might be expected given that they have been subjected to more intense selection than the other market classes, but there was significant admixture within those lines as evidenced by the high proportion of blue (subpopulation 3: cultivated tetraploids) within the Chip Processing subpopulation (subpopulation 1; Figure 2A, Table S1).

To test whether these results were due to the markers, the genotyping method, or the germplasm selected for the panel, STRUCTURE analysis was repeated using a subset of the markers, a subset of the lines, and the dosage based genotyping method (File S1). Collectively, these analyses supported the result that the only substructure within the panel was attributable to the inclusion of wild species and genetic stock lines, which are only a few generations removed from the wild species, rather than the cultivated potato market classes. Although there are defined market classes within cultivated potato (Chip Processing, French Fry Processing, Pigmented, Table Russet, Round White Table, and Yellow), many of these market classes are relatively recent. In addition, introgression crosses are used by breeding programs to introduce important agronomic traits such as disease resistance (Mackay 2005), which is reflected in the lack of observed structure within cultivated potato despite having subgroups of germplasm defined by the target market class.

Glycoalkaloid biosynthesis is a pathway common in solanaceous plants and is important for plant defense. However, glycoalkaloids can be toxic at high levels. In order to determine if genes in this pathway were selected during domestication and improvement, and are in part responsible for the separation of the wild species from cultivated potato in the STRUCTURE analysis, we analyzed 36 SNPs found within four candidate genes in the glycoalkaloid biosynthetic pathway. These included two genes involved in primary metabolism leading to cholesterol synthesis [*3-hydroxy-3-methylglutaryl coenzyme A reductase* (*HMG2*) and *squalene epoxidase* (*SQE*)] and two genes involved in secondary metabolism [*UDP-galactose:solanidine galactosyltransferase* (*SGT1*) and *UDP-glucose:solanidine glucosyltransferase* (*SGT2*)] (Ginzberg *et al.* 2012). Allele and genotype frequencies at these genes were no different in discriminating the cultivated potato clones from the wild species than were a similar number of randomly selected SNPs (Table S7). Therefore it is likely that the structure observed in Figure 2A does not reflect altered frequencies of SNPs in glycoalkaloid biosynthesis genes, but rather selection that may have occurred throughout the genome. However, introgression of novel alleles derived from wild species may not have been detected due to the origin of SNPs for the Infinium Array, which were derived exclusively from cultivated potato (Hamilton *et al.* 2011).

The finer resolution of pairwise genetic distances derived using an allele frequency- based (dosage) model revealed divergence within cultivated tetraploid market classes (Figure 2B, Figure S1). Interestingly, all of the market classes were concentrated on single branches with the exception of the Chip Processing market class, where three distinct groups of cold-sweetening resistant lines were identified (Lauer and Shaw 1970; Hamernik *et al.* 2009). One group was composed of lines derived from Lenape (Chip Processing Group I), which derives its cold-sweetening resistance from *S. chacoense* (Akeley *et al.* 1968). This is the oldest group of the Chip Processing lines, dating back to the 1960s. A second group consisted of relatives of ND860-2 (Chip Processing Group II), a grandchild of Lenape that also carries an introgression from *S. tuberosum* Group Phureja (Ehlenfeldt *et al.* 1990). The development of this group dates to the 1980s. The third group, which includes cultivars developed within the last decade, contained lines derived from S440 (Chip Processing Group III), which derives its cold-sweetening resistance from *S. tarijense* (Hermundstad 1986). Interestingly, the French Fry Processing and Table Russets were contained within a single branch, which suggests they were selected from a single russet-type germplasm pool. Indeed, although the French Fry Processing class has been selected for processing traits such as low reducing sugars and moderately high starch content and the Table Russets have been selected for appearance, both market classes have been selected for long tubers (1.75 ratio of length to width), russet skin, and similar optimum tuber size.

The clear trends of the market classes within the branches are indicative of the divergence in allelic content between the market classes. However, mixing of germplasm within the branches was observed to a limited extent. Some lines have characteristics of more than one market class (*i.e.*, yellow flesh and pigmented skin), which explains some of these inconsistencies. This mixing also is reflective of the culture and practice of potato breeding in the last century, where crossing outside of market classes has been used primarily to introgress disease resistance.

### Heterozygosity in cultivated potato *vs.* wild species

**Figure 3:**
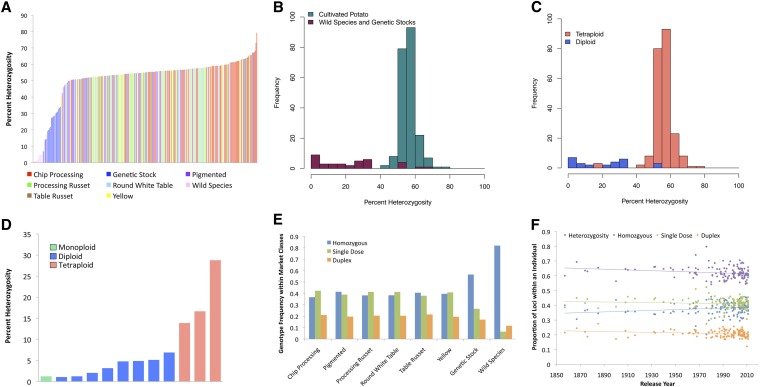
Percent heterozygosity and allele dosage frequency distributions. Percent heterozygosity was determined with 6373 SNP markers and a diploid genotyping model. Allele dosage frequencies were determined with 3763 SNP markers and a dosage genotyping model. (A) Distribution of percent heterozygosity across the 250 lines in the diversity panel. (B) Histogram of percent heterozygosity in cultivated potato *vs.* all of the wild species and genetic stocks. (C) Histogram of percent heterozygosity in diploid *vs.* tetraploid lines. (D) Percent heterozygosity in wild species individuals. (E) Distribution of average percent allele dosage frequencies within each market classes for tetraploid lines only. (F) Proportion of allele dosage frequencies within individual released lines by release year for tetraploid lines only.

In many crop species, generation of F_1_ hybrids has resulted in a yield advantage over inferior inbred parents (Brieger 1950; Duvick 2001), and potato breeders havelong theorized that maximum heterozygosity could lead to maximum heterosis (Mendoza and Haynes 1974). In the early 1960s, the maximum heterozygosity hypothesis was described in potato (Chase 1963), stating that maximum heterosis will be achieved by maximizing intra-locus diversity in autopolyploids. Using the diploid genotyping model and 6373 genome-wide SNPs, we found that the average percent heterozygosity observed within cultivated potato was 56%, with little observable variation among the market classes (Figure 3A). The percent heterozygosity in cultivated potato market classes ranged between 53% (Pigmented) and 59% (Chip Processing). In contrast, the Genetic Stocks had an average percent heterozygosity of 30%.

Wild and primitive cultivated *Solanum* species are often used in potato breeding programs because their extensive phenotypic diversity makes them valuable resources for high value traits (Mc**C**ann *et al.* 2010). Interestingly, the average percent heterozygosity observed was substantially lower in the samples of wild *Solanum* species examined compared to modern cultivated potato clones (Figure 3B). To correct for the sample size difference between the wild *Solanum* species and the cultivated potato clones, we selected 10,000 random subsamples of n = 12 from the cultivated potato clones. From the subsamples, the mean average percent heterozygosity was 56%, and the minimum was 51% (Figure S6), which was substantially greater than that observed for the 12 wild species. A comparison of diploid and tetraploid lines revealed substantially lower heterozygosity in diploids compared with tetraploids (Figure 3C) suggesting that greater ploidy may be associated with greater heterozygosity. For heterozygosity estimates in tetraploid germplasm, simplex, duplex, and triplex genotypic classes were reduced to a single heterozygous class. Direct tetraploid-to-tetraploid comparison can be made between *S. tuberosum* Group Andigena, the closest relative of modern cultivated potato, in which the heterozygosity of the one Andigena individual examined was only 29% (Figure 3D) compared to an average 56% in cultivated potato. For SNPs to be included on the Infinium SNP chip there could only be one additional SNP within a 100-bp window of the SNP (Hamilton *et al.* 2011) and as a consequence, the Infinium array represents genes with limited local polymorphism. Additionally, the SNPs were identified only from cultivated potato lines, although these lines contain introgressions from wild *Solanum* species. The simplification of tetraploid genotypes to a diploid model, the biallelic nature of the assay, assay design constraints, ascertainment bias, and sampling of a single individual from each wild *Solanum* species may have contributed to the differences in heterozygosity observed between wild species and cultivated potato. The magnitude of the reduction in heterozygosity in the wild *Solanum* species compared to cultivated potato measured with our 8303 Infinium SNP array was considerable, which raises fundamental questions about the genetic constitution of wild relatives of potato with regard to gene flow and population structure. However, the ascertainment bias and other limitations described above are likely to have underestimated polymorphism in the wild species. The use of an unbiased sampling method such as whole-genome shotgun sequencing of multiple lines from a panel of wild tuber-bearing *Solanum* species is likely to minimize these concerns and reveal more clearly the extent of genomic diversity in wild *vs.* cultivated potato.

Heterozygosity refers to having more than one allele at a locus. However, in a polyploid species like cultivated potato, there is an additional component, which is the dosage of those alleles over the four homologous chromosomes. The Infinium SNP array used in this study is a biallelic assay and as such cannot capture more than two distinct alleles at a locus. However, previous studies examining transcribed sequences in cultivated potato indicated that tri- and tetra-allelic variants at a single nucleotide level are rare (C. R. Buell and J. P. Hamilton, unpublished results). Thus, the biallelic nature of the assay should have limited effect on heterozygosity and allele dosage estimates in the transcribed regions of the potato genome. Across the cultivated potato market classes, little variation was observed in the frequency of homozygous (AAAA or BBBB), single dose (ABBB or AAAB), and duplex (AABB) loci, with a ratio of approximately 2:2:1 for homozygous/single dose/duplex (Figure 3E). In contrast, the ratio of single dose to duplex loci was much closer to 1:1 in the tetraploid Genetic Stocks, which represent an intermediate between the wild species and cultivated potato. The diversity panel contains historical lines released as early as 1857 and modern cultivars released as recently as 2011, with little variation in allele dosage observed across decades (Figure 3F). These data suggest that heterozygosity on a genome-wide scale was achieved prior to formal breeding efforts in the mid 1800s, and that potato breeding during the last century resulted in changes to allele composition at select loci to address the needs of specific market classes rather than genome-scale alterations of heterozygosity. Overall, this genome-wide assessment of diversity suggests that through ~150 years of breeding, including intensive breeding in the Chip and French Fry Processing market classes, substantial changes in heterozygosity and allele dosage have not occurred. However, as described in the sections to follow, clear selection was apparent for alleles in biosynthetic pathways that are integral to phenotypic traits such as pigmentation or carbohydrate amount and composition.

### Selection for high carotenoid potatoes

Potatoes with yellow flesh contain elevated amounts of the carotenoids α-carotene and β-carotene, which are the main precursors of vitamin A. Vitamin A is important for immunity, eye function, and as an antioxidant, among other health benefits. The carotenoid biosynthetic pathway in potato has been well described (Diretto *et al.* 2006). Lycopene is produced from phytoene by the action of *phytoene dehydrogenase* (*PDS*) and *zeta-carotene desaturase* (*ZDS*) and is the substrate for *lycopene epsilon cyclase* (*LCY-e*) and *lycopene beta cyclase* (*LCY-b*), which produce α-carotene and β-carotene. Silencing of *LCY-e* results in elevated β-carotene accumulation (Diretto *et al.* 2006), a potential target for engineering potatoes with increased carotenoid content.

**Figure 4:**
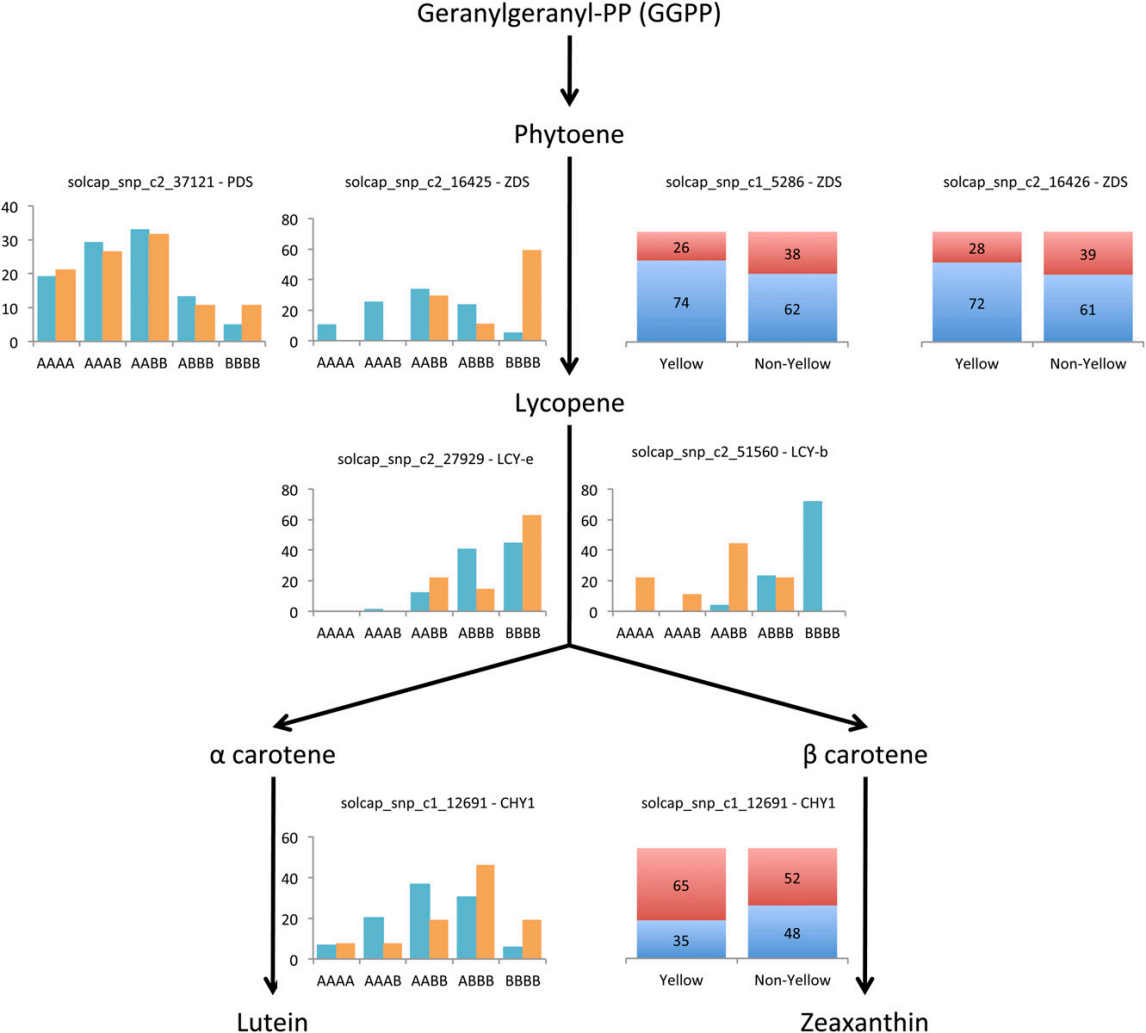
Selection for carotenoid biosynthetic pathway genes in Yellow fleshed market class potato lines. SNPs located within carotenoid biosynthetic pathway genes were tested for significant differences in allele or genotype composition compared to that observed in all other cultivated potato lines, and only SNPs that had a *P* < 0.05 for either genotype or allele frequencies are shown. In some cases, a SNP was only significant at either the genotype or allele level. Orange bars indicate percent of the Yellow lines with a given genotype, and aqua bars indicate percent of all other cultivated tetraploid potato lines. Blue and red bars indicate the percent of A and B alleles, respectively. *PDS*, *phytoene dehydrogenase*; *ZDS*, *zeta-carotene desaturase*; *LCY-e*, l*ycopene epsilon cyclase*; *LCY-b*, *lycopene beta cyclase*; *CHY1*, *beta-carotene hydroxylase 1*.

Selection for the Yellow market class in the United States has only occurred in the past 30 years. SNPs in several genes in the carotenoid biosynthetic pathway, including *LCY-e* and *LCY-b*, were present on the Infinium array. These SNPs were tested for differences in genotype and allele frequency between the Yellow market class and the other cultivated market classes that are not enriched for carotenoids. For the five SNPs with a significant difference in genotype frequency, selection toward enrichment of a specific homozygous class (AAAA or BBBB) was observed in Yellow lines relative to other cultivated potatoes (Figure 4, Table S5, Table S10). Changes in overall allele frequency were observed in SNPs for *ZDS* and *beta-carotene hydroxylase 1* (*CHY1*) (Figure 4, Table S5, Table S10). Analyses using a knockout approach previously identified *CHY1* as significantly effecting yellow tuber flesh color (Diretto *et al.* 2007). In addition, naturally occurring variation in carotenoid content has been linked to *CHY1* alleles as well (Brown *et al.* 2006). Similarly, the importance of *LCY-b* expression (Ampomah-Dwamena *et al.* 2009) and *ZDS* allelic composition (Wong *et al.* 2004) has previously been shown to be important for carotenoid accumulation in other species. Three of the five SNPs that showed significant differences in genotype or allele frequency resulted in a non-synonymous change (data not shown). Despite the short time over which selection has occurred for the Yellow market class in the United States, major changes in allele and genotype frequencies for carotenoid biosynthetic pathway genes have been observed. These results highlight the utility and power of genome-scale studies in guiding selection in breeding programs.

### Selection for improved processing potatoes

Potato chips were invented in the 1850s, but until the 1950s, potato breeding efforts focused exclusively on the fresh market class with chips made using the best material available locally. However, the chip-processing industry in the United States expanded dramatically after World War II and breeders, working with potato chip manufacturers, began to select for the traits most important to that industry, including high specific gravity (starch) and low reducing sugar content of tubers. Cultivars bred specifically for chipping began to be released in the 1960s with early chip cultivars, including Monona (Stevenson *et al.* 1965), Lenape (Akeley *et al.* 1968), Wauseon (Cunningham *et al.* 1968), and Norchip (Johansen *et al.* 1969).

**Figure 5:**
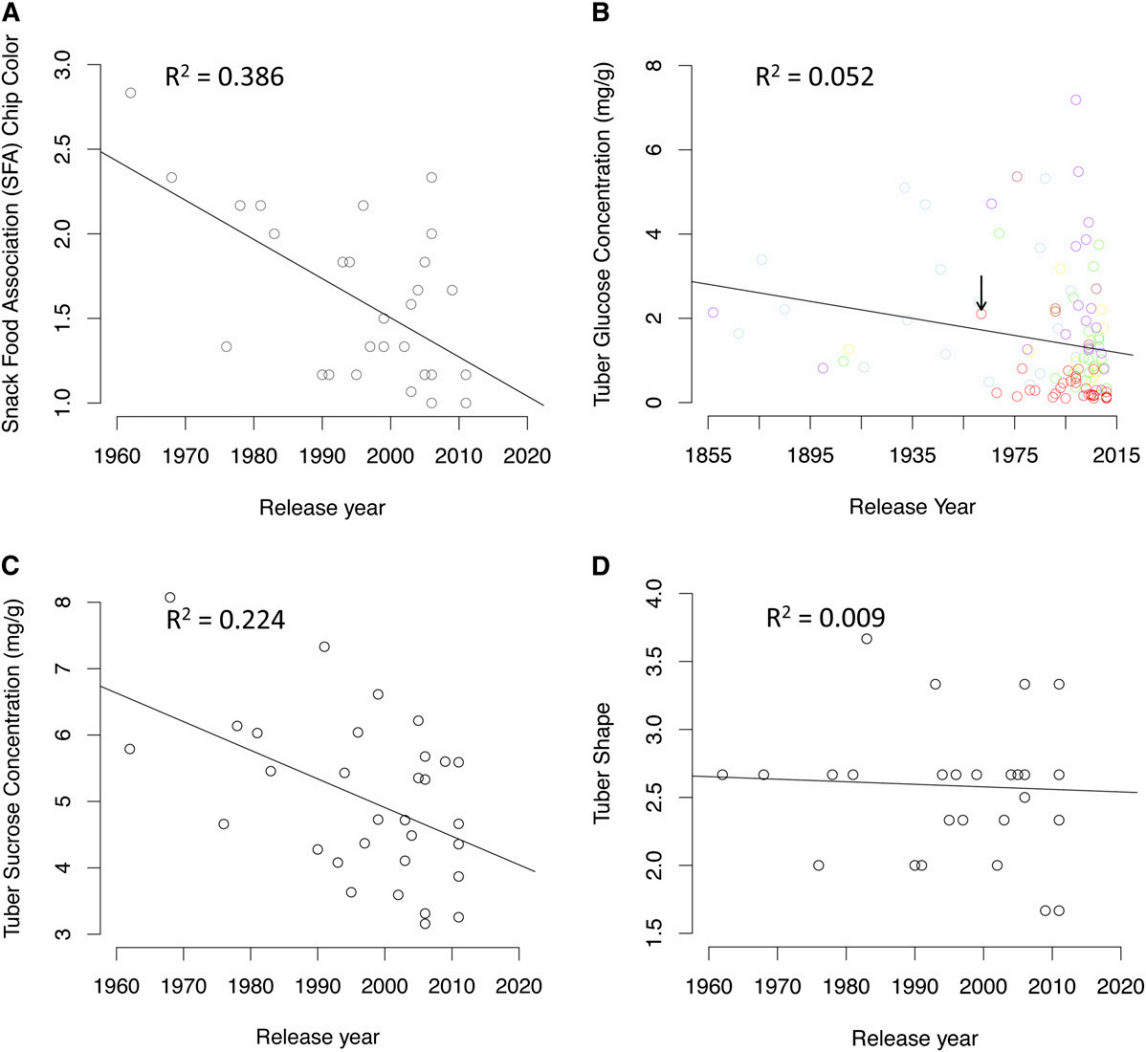
Effect of phenotypic selection on market class divergence and overall species improvement over a century of potato breeding. Phenotypic traits were evaluated in three total replications in 2010 across two locations (New York and Wisconsin). (A) Scatter plot of least square means by release year for 31 potato chip lines that have been released between 1962 and 2011. Low snack food association chip color corresponds to lighter chip color, and 2.0 is considered acceptable by the potato chip industry. (B) Scatter plot of least square means by release year for the 115 lines in the diversity panel that have been released between 1857 and 2011. Lines are color-coded based on market class (Chip Processing: red, French Fry Processing: green, Pigmented: purple, Round White Table: light blue, Table Russet: brown, and Yellow: yellow). The black arrow is pointing to La Chipper. (C and D) Scatter plots of least square means by release year for 31 potato chip lines that have been released between 1962 and 2011.

The need for potatoes with physical and biochemical properties that specifically match industry requirements for the production of chips resulted in highly focused phenotypic selection within the Chip Processing market class. A major indicator of chip quality is color after frying measured on the SFA chip color scale, with lighter color being highly desirable (Figure S3). In this regard, there has been remarkable improvement during the past 50 years, beginning with the early cultivars specifically bred for chipping and continuing with present lines (Figure 5A). Dark chip color is produced during frying from the reducing sugars glucose and fructose, both of which are formed predominantly from the hydrolysis of tuber sucrose. Tuber glucose concentration, which was directly selected against due to its effect on chip color, was dramatically reduced in the late 1960s with the advent of the Chip Processing class, and has seen modest reductions since that time (Figure 5B). La Chipper, released in 1962 and perhaps the first chip cultivar developed in the U.S., was a notable exception to this. A consistent decrease in tuber sucrose content has also been achieved over time (Figure 5C), and this may reflect indirect selection for decreased reducing sugar content and increased duration of storage. In contrast, little or no change was observed for tuber shape over time within the Chip Processing clones since round tubers are preferred for processing and do not have the strict length to width ratio demanded by the French fry processing industry (Figure 5D).

**Figure 6:**
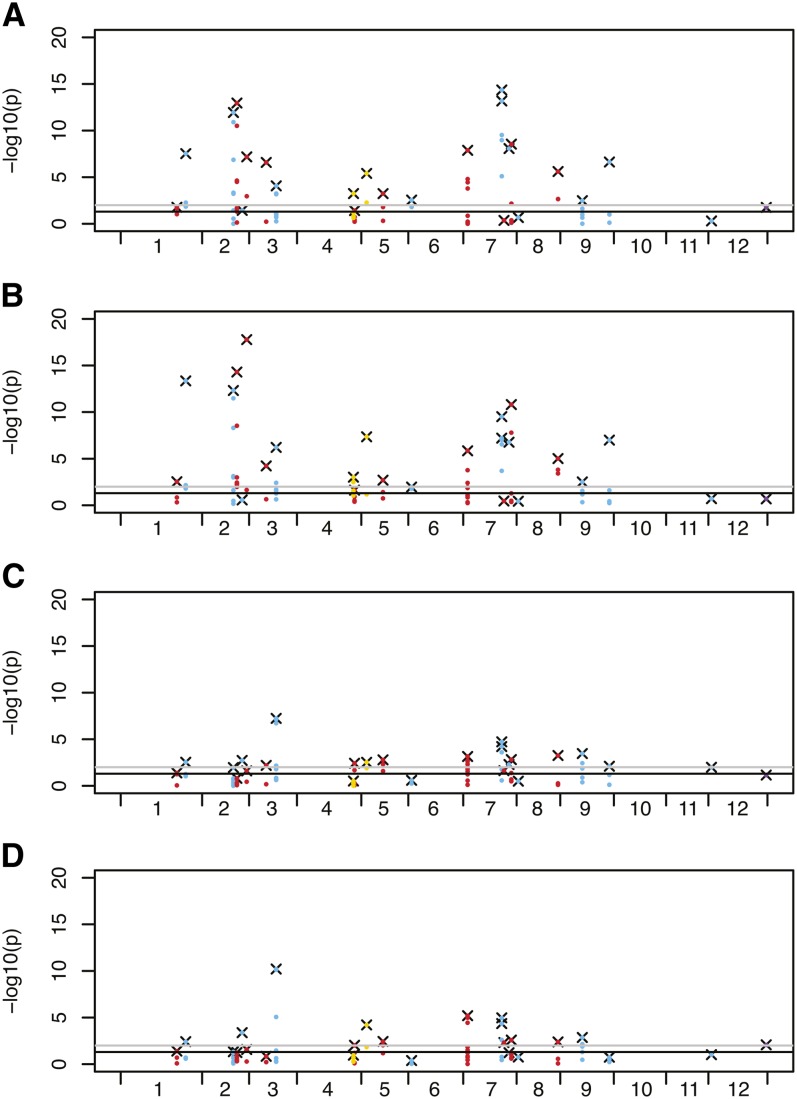
Selection for carbohydrate synthesis, degradation, transport, and regulatory genes in Chip Processing clones. SNPs within 25 genes were tested for significant differences in allele and genotype composition between Chip Processing clones and all other cultivated potato [(A) allele, (B) genotype] and between French Fry Processing clones and non-processing cultivated potato clones [Pigmented, Round White Table, Table Russet, Yellow; (C) allele, (D) genotype]. The x-axis in all graphs is the position on each of the 12 potato chromosomes and the y-axis is a –log10 transformation of the *P*-value for the χ^2^ tests. Blue data points are SNPs in carbohydrate degradation genes, red data points are SNPs in carbohydrate synthesis genes, yellow data points are SNPs in carbohydrate transport genes, and the purple data point is a SNP in a regulatory gene annotated as invertase inhibitor. The data points with an x behind them are the most significant SNP within a gene. The black horizontal lines indicate significance at *P* = 0.05 and the gray lines at *P* = 0.01.

SNPs were evaluated in 25 genes associated with carbohydrate synthesis, degradation, regulation, and transport were evaluated and compared to a set of randomly selected SNPs occurring on ten chromosomes and an unanchored scaffold (Table S6). A comparison of SNPs in Chip Processing clones with all other cultivated lines revealed that breeders have significantly altered most of the carbohydrate-related genes in this market class with respect to the allelic (Figure 6A) and genotypic (Figure 6B) frequency relative to changes observed in randomly selected genes. Multiple SNPs in starch accumulation genes (*sucrose synthase*, *sucrose synthase 2*), starch synthesis genes (*granule bound starch synthase 1*, *granule bound starch synthase 2*, *soluble starch synthase*), and starch breakdown genes (*isoamylase isoform 1*) have been strongly enriched (*P* < 0.01) in the Chip Processing class relative to all other cultivated lines.

Color after frying is also important in the French Fry Processing lines. However, the French fry processing industry uses different methods, such as blanching, which reduce the effects of reducing sugars on the appearance of the final product. Thus, we hypothesized that there would be less differentiation in carbohydrate synthesis, degradation, regulation, and transport genes in the French Fry Processing lines compared with the Chip Processing lines. Allelic and genotypic frequency differences in many of the 25 genes were also observed in lines selected for French fry production when compared to lines that have not been selected for processing (Figure 6, C and D and Table S6); however, fewer significant alterations in SNP frequencies were observed compared to the Chip Processing lines, and *P*-values associated with altered SNPs tended to be less significant in the French Fry Processing comparisons (Figure 6). For both allelic and genotypic frequencies, 62% of the carbohydrate candidate gene SNPs differentiated Chip Processing lines from other cultivated potato lines compared with 47% (allelic) and 35% (genotypic) of randomly selected SNPs. While, for the French Fry processing lines compared with the nonprocessing cultivated potato lines, 41% and 34% of the carbohydrate candidate gene SNPs were discriminatory for allelic and genotypic frequencies respectively, compared to 53% (allelic) and 24% (genotypic) of randomly selected SNPs. It is interesting that multiple SNPs in the genes encoding *β-amylase* on chromosome 1, *granule-bound starch synthase* on chromosomes 2 and 8, *Isoamylase isoforms 1 and 3*, *pyruvate decarboxylase*, and *soluble starch synthase* have been enriched in the Chip Processing lines much more than in the French Fry Processing lines. This is particularly notable for *soluble starch synthase*, where 10 of 11 SNPs within the gene had altered allelic frequency and 11 of 11 had altered genotypic frequency in the Chip Processing lines, whereas none of those SNPs were altered in the French Fry Processing lines. It is likely that more intense selection for the high starch content and low reducing sugar content required in processing of the Chip Processing lines *vs.* the French Fry Processing lines has contributed to these differences.

## Conclusions

The modern era of potato breeding has focused primarily on phenotypic selection to improve agronomic quality, yield, and market class−specific traits. Potato breeding in the 1950s expanded to include intensive germplasm introgression and strategies involving ploidy manipulation and species introgression to broaden the genetic base and improve new market traits especially for the processing market classes. With the release of the potato genome sequence and subsequent genomic tools, as well as a rich collection of historic potato lines, we were able to retrospectively examine the effect of phenotypic selection on the potato genome. STRUCTURE analysis showed clear differences between the cultivated potato clones and the related wild species and minimal substructure within cultivated potato, despite the introgression of wild species into cultivated potato and selection for market class specific lines. Separation of the cultivated potato market classes was observed using the allele frequency based model to determine pair-wise genetic distances.

The maximum heterozygosity theory has driven breeders to use methods to increase heterozygosity within cultivated potato, such as making wide crosses with wild *Solanum* relatives. Our genome-wide survey of diversity in cultivated potato, genetic stocks, and wild *Solanum* species revealed significantly more heterozygosity in cultivated potato compared to wild *Solanum* species. The low heterozygosity in the majority of wild species was surprising, suggesting a need to use species-wide diversity rather than individual accessions to increase allelic diversity in cultivated potato. Interestingly, despite concentrated efforts, heterozygosity in cultivated potato remained unchanged over ~150 years of breeding. These results may reflect the limited number of breeding cycles that have been completed owing to the decade of testing required within a breeding cycle. Additionally, the analysis of heterozygosity was conducted at the individual base pair level rather than the haplotype level. Changes in functionally important heterozygosity may only be detectable at the gene/haplotype level.

Although genome-scale changes in heterozygosity were not observed within cultivated lines, there has been clear selection of alleles for traits relevant to specific market classes. Significant differences in genotype and allele content between the Yellow market class and all other cultivated potato market classes was observed for multiple steps in the carotenoid biochemical pathway. Additionally, for both processing market classes (Chip Processing and French Fry Processing), significant differences relative to other cultivated potato market classes were observed for SNPs in carbohydrate synthesis, degradation, regulation, and transport genes. Interestingly, more SNPs with significant differences were observed in the Chip Processing market class comparisons than the French Fry Processing market class comparisons likely due to the differences in requirements set by the two postharvest processing industries.

Selection for alleles in carotenoid biosynthesis and carbohydrate metabolism relevant to market classes demonstrates the success of potato breeders during the last 50 years in improving the nutritional and processing traits in cultivated potato. However, an increased rate of gain from selection over time will be needed to meet worldwide food demands and challenges due to climate change, including higher yields, abiotic stress tolerance, and disease resistance. Potato breeding efforts, which have historically been based on phenotypic selection, would benefit from marker-assisted selection and more opportunities to shuffle the genome to create new variation. The genomic tools are now in place to identify associations between genotype and phenotype and improve future efforts in potato breeding.

## Supplementary Material

Supporting Information
