# Supplementary material for: Retrospective View of North American Potato (Solanum tuberosum L.) Breeding in the 20th and 21st Centuries
Source: G3 (Bethesda). 2013 Jun 1;3(6):1003–13. doi: 10.1534/g3.113.005595 (PMC3689798; doi:10.1534/g3.113.005595)
Supplement: Supporting Information [file supp_3_6_1003__index.html]

Retrospective View of North American Potato (Solanum tuberosum L.) Breeding in the 20th and 21st Centuries — Supporting Information 

# Retrospective View of North American Potato (*Solanum tuberosum* L.) Breeding in the 20th and 21st Centuries

## Supporting Information for Hirsch *et al.*, 2013

**Files in this Data Supplement:**

- Supporting Information - Figures S1-S6, File S1, and Tables S1-S10 (PDF, 7 MB)
- Figure S1 - Unweighted pair group method with arithmetic mean (UPGMA) tree of 250 potato lines based on 3,763 single nucleotide polymorphism markers with dosage genotype calls and individual lines labeled (PDF, 685 KB)
- Figure S2 - Workflow used to determine dosage genotype scores (PDF, 78 KB)
- Figure S3 - Snack Food Association (SFA) chip color measurement standard (PDF, 4 MB)
- Figure S4 - Tuber shape measurement standard (PDF, 1 MB)
- Figure S5 - Average likelihood estimates from STRUCTURE with varying *K* (number of populations) values (PDF, 106 KB)
- Figure S6 - Histogram of average percent heterozygosity in subsamples (N=12) of the cultivated potato clones (PDF, 66 KB)
- File S1 - Summary of STRUCTURE validation testing the effects of varying marker number, genotype method, and germplasm included in the analysis (PDF, 60 KB)
- Table S5 - Single nucleotide polymorphisms (SNPs) in carotenoid biosynthetic pathway genes and random SNPs tested for significant differences in allele or genotype composition compared to that observed in all other cultivated potato lines (PDF, 66 KB)
- Table S7 - Single nucleotide polymorphisms (SNPs) in glycoalkaloid biosynthetic pathway candidate genes and random SNPs throughout the genome tested for significant differences in allele or genotype composition between the Wild Species and cultivated potato lines (PDF, 72 KB)
- Table S8 - Significance of model effects for the 190 tetraploid lines with phenotypic data (PDF, 59 KB)
- Table S9 - Least square means of 190 tetraploid lines with phenotypic data (PDF, 92 KB)
- Table S10 - Summary of single nucleotide polymorphisms (SNPs) in carotenoid biosynthetic pathway genes tested for significant differences in allele or genotype composition compared to that observed in all other cultivated potato lines (PDF, 60 KB)
- Table S1 - Species, population structure grouping, market class, ploidy level, and release year information for 250 diverse potato lines (.xlsx, 55 KB)
- Table S2 - Dosage cluster calling boundaries for the Potato 8,303 Infinium SNP Chip for the 5,031 SNPs whose dosage boundaries could be determined (.xlsx, 352 KB)
- Table S3 - Genotype scores for 6,373 single nucleotide polymorphism markers on 250 lines using a diploid model (AA, AB, BB) (.xlsx, 5 MB)
- Table S4 - Genotype scores for 3,763 single nucleotide polymorphism markers on 250 lines using a dosage model (monoploid A and B; diploid AA, AB, and BB; tetraploid AAAA, AAAB, AABB, ABBB, and BBBB) (.xlsx, 3 MB)
- Table S6 - Single nucleotide polymorphisms (SNPs) in carbohydrate degradation, synthesis, transport, and regulation genes tested for significant differences in allele or genotype composition compared to that observed in all other cultivated potato lines (.xlsx, 81 KB)
