# Supplementary material for: Retrospective View of North American Potato (Solanum tuberosum L.) Breeding in the 20th and 21st Centuries
Source: G3 (Bethesda). 2013 Jun 1;3(6):1003–13. doi: 10.1534/g3.113.005595 (PMC3689798; doi:10.1534/g3.113.005595)
Supplement: Supporting Information [file supp_g3.113.005595_005595SI.pdf]

G3 · Genes | Genomes | Genetics

Retrospective View of North American Potato (*Solanum tuberosum* L.) Breeding in the 20th and 21st Centuries

Candice N. Hirsch[*], Cory D. Hirsch[*], Kimberly Felcher[§], Joseph Coombs[§], Dan Zarka[§], Allen Van Deynze[†], Walter De Jong[‡], Richard E. Veilleux[**], Shelley Jansky[§§,††], Paul Bethke[§§,††], David S. Douches[§], C. Robin Buell[*].

[*]Department of Plant Biology, Michigan State University, East Lansing, MI, 48824
[§]Department of Plant, Soil and Microbial Sciences, Michigan State University, East Lansing, MI, 48824
[†]Seed Biotechnology Center, University of California, Davis, CA, 95616
[‡]Department of Plant Breeding & Genetics, Cornell University, Ithaca, NY, 14853
[**]Department of Horticulture, Virginia Polytechnic Institute and State University, Blacksburg, VA, 24061
[§§]Department of Horticulture, University of Wisconsin, Madison, WI, 53706
[††]United States Department of Agriculture-Agricultural Research Service, Vegetable Crops Research Unit, Madison, Wisconsin, 53706

DOI: 10.1534/g3.113.005595

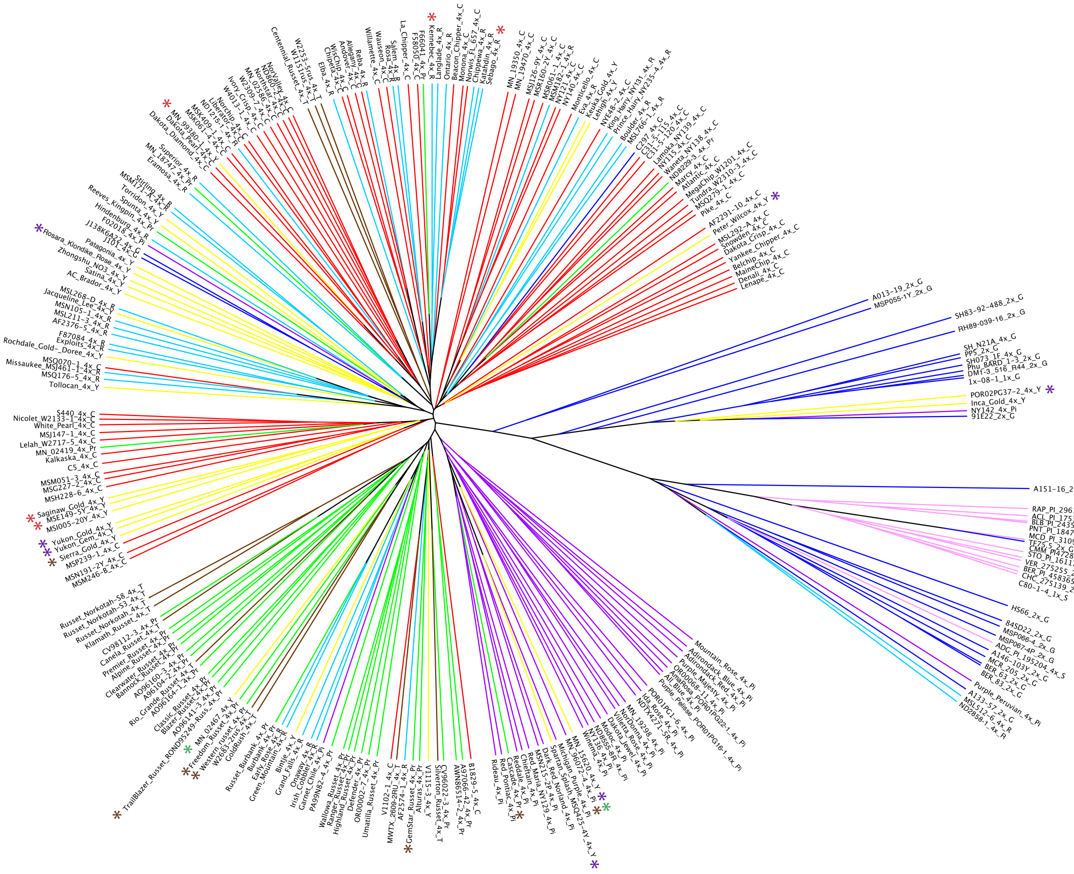

Figure S1 Unweighted pair group method with arithmetic mean (UPGMA) tree of 250 potato lines based on 3,763 single nucleotide polymorphism markers with dosage genotype calls and individual lines labeled. Color-coding is based on predominant market class designation and asterisks indicate lines that can be classified into more than one market class (red – Chip Processing; dark blue – Genetic Stock; purple – Pigmented; green - French Fry Processing; light blue – Round White Table; pink – Wild Species; brown – Table Russet; yellow – Yellow).

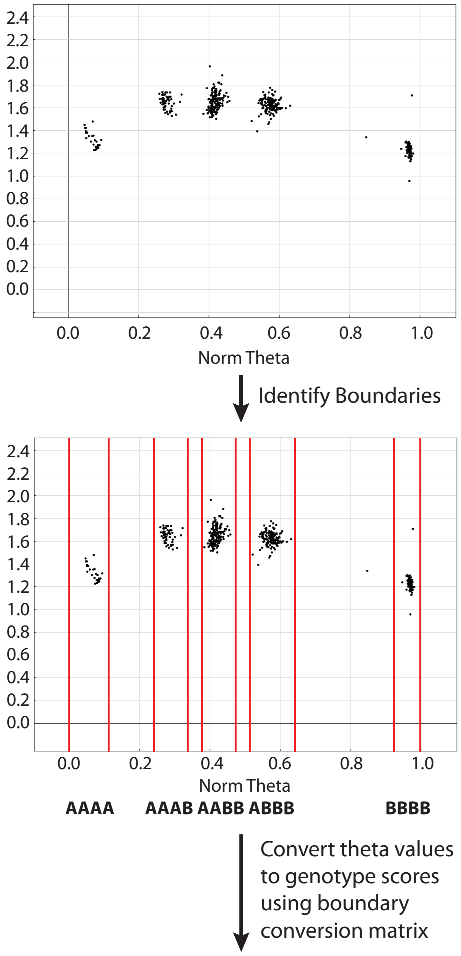

	Clone1	Clone2	Clone3	Clone4	Clone5
SNP1	AABB	ABBB	AAAA	ABBB	BBBB
SNP2	BBBB	ABBB	ABBB	AAAA	ABBB
SNP3	ABBB	AAAA	ABBB	ABBB	BBBB
SNP4	AABB	ABBB	AAAA	ABBB	ABBB
SNP5	AABB	ABBB	AAAA	AAAA	AABB
SNP6	AAAA	ABBB	BBBB	ABBB	AABB

Figure S2 Workflow used to determine dosage genotype scores.

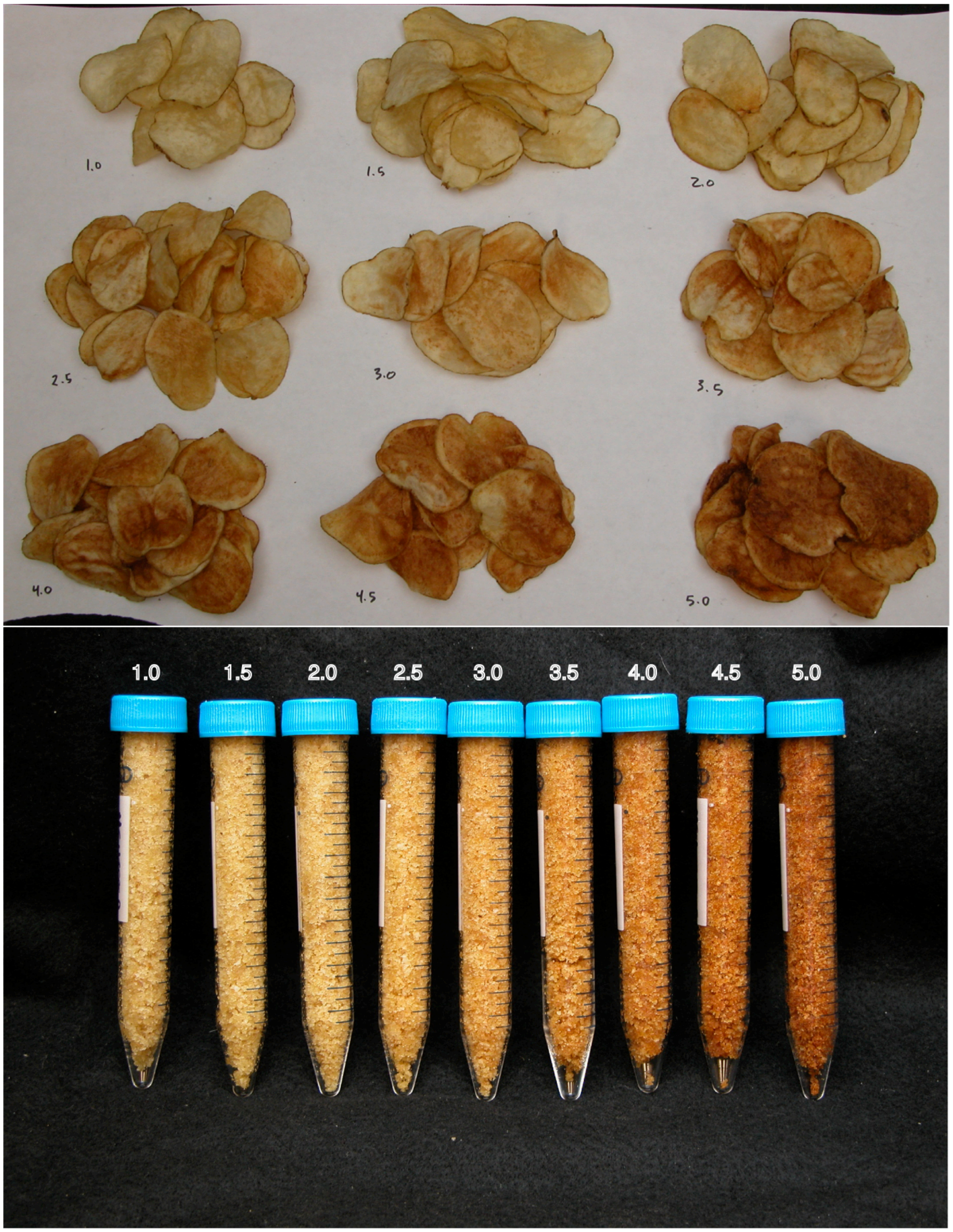

Figure S3 Snack Food Association (SFA) chip color measurement standard.

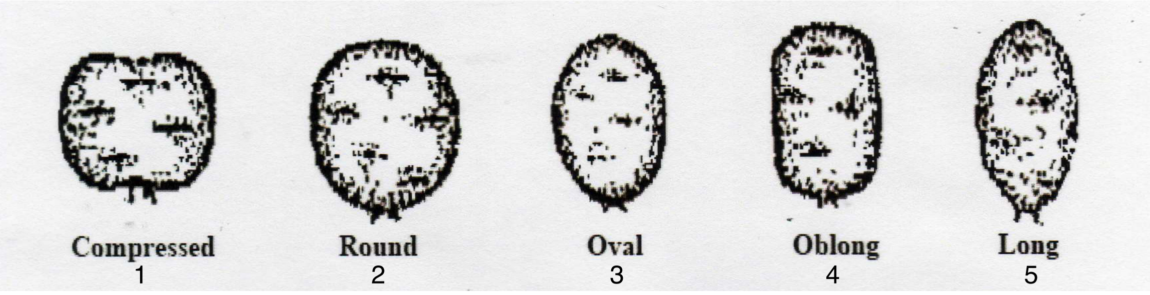

Figure S4 Tuber shape measurement standard.

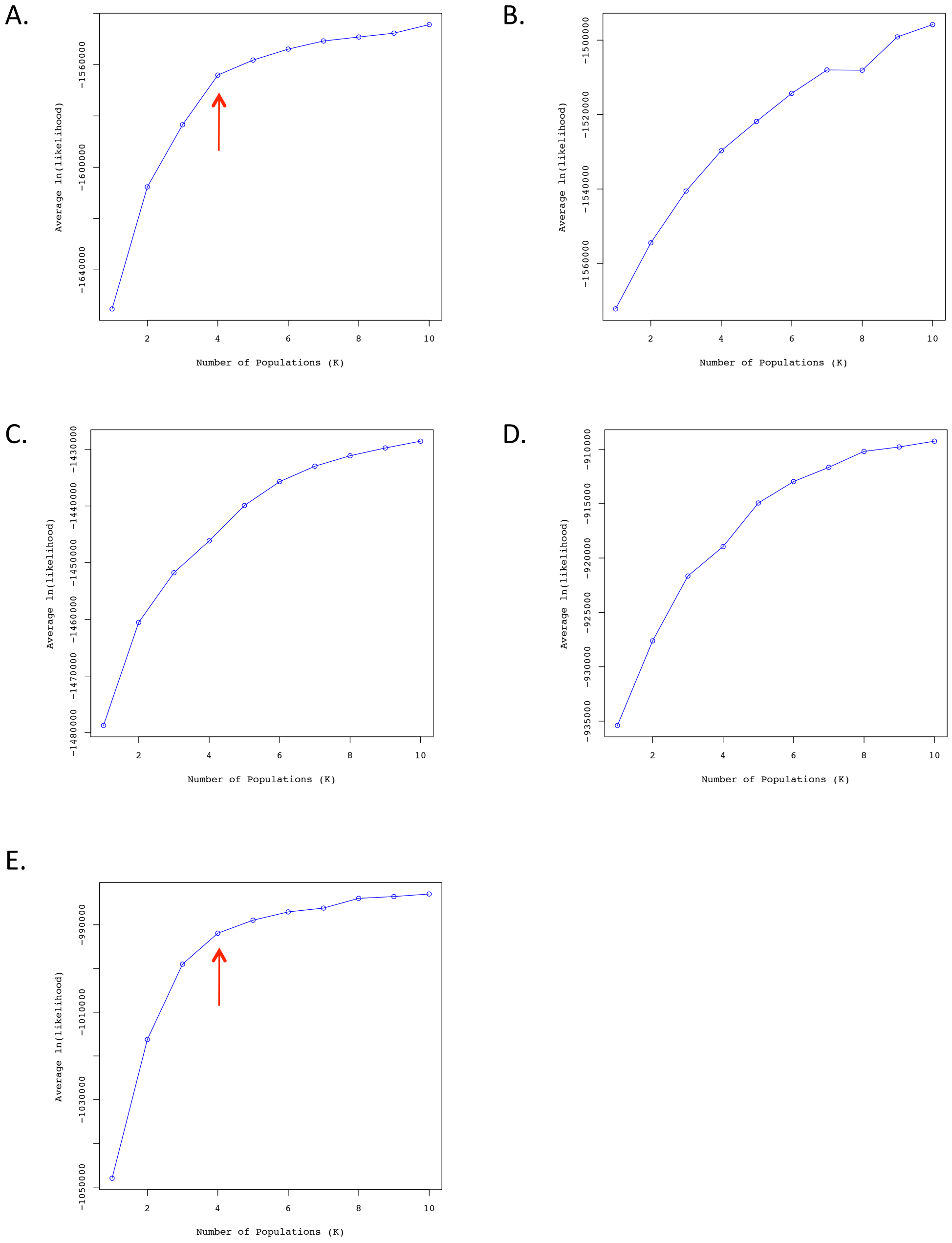

Figure S5 Average likelihood estimates from STRUCTURE with varying *K* (number of populations) values. STRUCTURE was run on multiple datasets, and likelihood estimates are shown for (A) diploid dataset, (B) tetraploid as tetraploid dataset, (C) tetraploid as diploid dataset, (D) tetraploid as diploid subset, and (E) diploid subset. The red arrows indicate the inflection points in each graph if a clear inflection point was observed.

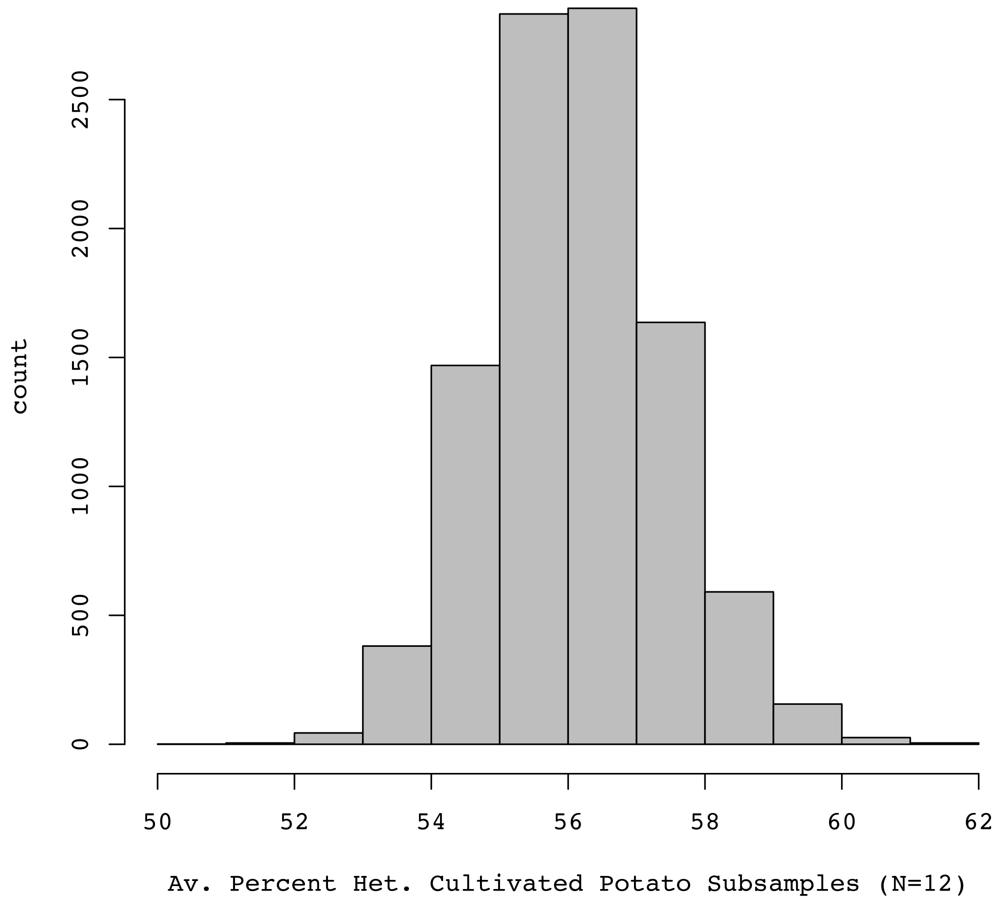

Figure S6 Histogram of average percent heterozygosity in subsamples (N=12) of the cultivated potato clones. Sub-sampling was performed with replacement 10,000 times.

File S1 Summary of STRUCTURE validation testing the effects of varying marker number, genotype method, and germplasm included in the analysis.

STRUCTURE (Pritchard *et al.* 2000) analysis was conducted with only the tetraploid clones (221 clones) using the dosage genotyping model (AAAA, AAAB, AABB, ABBB, BBBB; 3,763 markers) to evaluate if subpopulations exist within cultivated potato that could not be resolved due to the inclusion of Genetic Stock and Wild Species in the previous analysis. However, no inflection point was observed in the maximum likelihood graphs with a maximum *K* value of 10 (Figure S5B). In addition to removing the Genetic Stock and Wild Species, this analysis used a subset of the markers and dosage genotyping calls. To confirm that the lack of observed structure was not from either of these variables, analysis of the tetraploid clones was repeated using the diploid genotyping model (AA, AB, BB) with all of the markers that could be scored (6,373 markers; Figure S5C) and with the subset of markers that dosage genotyping could be called on (3,763 markers; Figure S5D) and again no inflection point was observed. However, repeating the analysis with the entire panel (250 clones) using the diploid genotyping model but only with the subset of markers that dosage genotyping could be called on (3,763 markers) a clear inflection point was again observed for *K*=4 (Figure S5E).

Pritchard, J. K., M. Stephens and P. Donnelly, 2000 Inference of population structure using multilocus genotype data. Genetics **155:** 945-959.

Table S1 Species, population structure grouping, market class, ploidy level, and release year information for 250 diverse potato lines. Population structure was determined using STRUCTURE with 6,373 SNP markers.

Table S1 is available for download as a xlsx file at
http://www.g3journal.org/lookup/suppl/doi:10.1534/g3.113.005595/-/DC.

Table S2 Dosage cluster calling boundaries for the Potato 8,303 Infinium SNP Chip for the 5,031 SNPs whose dosage boundaries could be determined. NA indicates that the boundary for a specific marker class (AAAA, ABBB, AABB, ABBB, BBBB) could not be clearly determined.

Table S2 is available for download as a XLSX file at http://www.g3journal.org/lookup/suppl/doi:10.1534/g3.113.005595/-/DC.

Table S3 Genotype scores for 6,373 single nucleotide polymorphism markers on 250 lines using a diploid model (AA, AB, BB).

Table S3 is available for download as a XLSX file at http://www.g3journal.org/lookup/suppl/doi:10.1534/g3.113.005595/-/DC.

Table S4 Genotype scores for 3,763 single nucleotide polymorphism markers on 250 lines using a dosage model (monoploid – A and B; diploid AA, AB, and BB; tetraploid AAAA, AAAB, AABB, ABBB, and BBBB).

Table S4 is available for download as a XLSX file at http://www.g3journal.org/lookup/suppl/doi:10.1534/g3.113.005595/-/DC.

Table S5 Single nucleotide polymorphisms (SNPs) in carotenoid biosynthetic pathway genes and random SNPs tested for significant differences in allele or genotype composition compared to that observed in all other cultivated potato lines. Bold numbers indicate significance at p-value≤0.05.

SNP Type	SNP Name	PGSC Gene	Annotation	Allele Frequency p-value	Genotype Frequency p-value
Carotenoid	solcap_snp_c2_13431	PGSC0003DMG400013335	alternative oxidase	0.503	0.622
Carotenoid	solcap_snp_c1_12691	PGSC0003DMG400028897	beta-carotene hydroxylase	**0.006**	**0.005**
Carotenoid	solcap_snp_c2_45240	PGSC0003DMG400008159	lycopene beta cyclase, chloroplastic	0.976	0.886
Carotenoid	solcap_snp_c2_45242	PGSC0003DMG400008159	lycopene beta cyclase, chloroplastic	0.917	0.648
Carotenoid	solcap_snp_c2_45244	PGSC0003DMG400008159	lycopene beta cyclase, chloroplastic	0.875	0.668
Carotenoid	solcap_snp_c2_51559	PGSC0003DMG400010637	lycopene beta cyclase, chloroplastic	0.802	0.222
Carotenoid	solcap_snp_c2_51560	PGSC0003DMG400010637	lycopene beta cyclase, chloroplastic	0.220	**0.045**
Carotenoid	solcap_snp_c2_37118	PGSC0003DMG400009156	phytoene dehydrogenase, chloroplastic/chromoplastic	0.345	0.195
Carotenoid	solcap_snp_c2_37121	PGSC0003DMG400009156	phytoene dehydrogenase, chloroplastic/chromoplastic	0.070	**0.035**
Carotenoid	solcap_snp_c1_9060	PGSC0003DMG400028224	carotenoid isomerase, chloroplastic	0.131	0.417
Carotenoid	solcap_snp_c2_29741	PGSC0003DMG400028224	carotenoid isomerase, chloroplastic	0.589	0.051
Carotenoid	solcap_snp_c1_5286	PGSC0003DMG400022473	zeta-carotene desaturase	**0.010**	0.239
Carotenoid	solcap_snp_c2_16424	PGSC0003DMG400022473	zeta-carotene desaturase	0.236	0.070
Carotenoid	solcap_snp_c2_16425	PGSC0003DMG400022473	zeta-carotene desaturase	0.217	**0.011**
Carotenoid	solcap_snp_c2_16426	PGSC0003DMG400022473	zeta-carotene desaturase	**0.019**	0.300
Carotenoid	solcap_snp_c2_27926	PGSC0003DMG400000333	lycopene epsilon cyclase	0.687	0.723
Carotenoid	solcap_snp_c2_27929	PGSC0003DMG400000333	lycopene epsilon cyclase	0.438	**0.031**
Carotenoid	solcap_snp_c2_27930	PGSC0003DMG400000333	lycopene epsilon cyclase	0.512	0.363
Random	solcap_snp_c1_13189	PGSC0003DMG400022953	pyruvate decarboxylase	0.370	0.360
Random	solcap_snp_c1_4990	PGSC0003DMG400010454	transitional endoplasmic reticulum ATPase	0.356	0.123
Random	solcap_snp_c2_11479	PGSC0003DMG400027738	GTP-binding protein	0.829	0.384
Random	solcap_snp_c2_11758	PGSC0003DMG400000810	serine-threonine protein kinase, plant-type	0.604	0.524
Random	solcap_snp_c2_15332	PGSC0003DMG400008081	auxin response factor	0.171	0.326
Random	solcap_snp_c2_15342	PGSC0003DMG400008081	auxin response factor	0.122	0.313
Random	solcap_snp_c2_19537	PGSC0003DMG400013943	conserved gene of unknown function	0.194	**0.032**
Random	solcap_snp_c2_22003	PGSC0003DMG400032243	homocysteine s-methyltransferase	**0.017**	0.065
Random	solcap_snp_c2_23942	PGSC0003DMG400000994	calcium-dependent protein kinase 3	0.925	0.222
Random	solcap_snp_c2_34	-	-	0.527	0.740
Random	solcap_snp_c2_35055	PGSC0003DMG400009380	receptor protein kinase CLAVATA1	**0.017**	**0.020**
Random	solcap_snp_c2_36264	PGSC0003DMG400013391	conserved gene of unknown function	0.721	0.792
Random	solcap_snp_c2_46455	PGSC0003DMG400025176	non-symbiotic hemoglogin	0.908	0.474
Random	solcap_snp_c2_55863	PGSC0003DMG400023897	self-pruning G-box protein	0.079	0.562
Random	solcap_snp_c2_9003	-	-	0.267	0.847
Random	solcap_snp_c1_15926	PGSC0003DMG400022947	uncharacterized aarF domain-containing protein kinase, chloroplastic	**0.003**	0.062
Random	solcap_snp_c2_20569	PGSC0003DMG400028985	calcium ion binding protein	0.849	0.262
Random	solcap_snp_c2_49451	PGSC0003DMG400001685	acyl carrier protein	0.252	0.296

Table S6 Single nucleotide polymorphisms (SNPs) in carbohydrate degradation, synthesis, transport, and regulation genes tested for significant differences in allele or genotype composition compared to that observed in all other cultivated potato lines.

Table S6 is available for download as a XLSX file at http://www.g3journal.org/lookup/suppl/doi:10.1534/g3.113.005595/-/DC.

Table S7 Single nucleotide polymorphisms (SNPs) in glycoalkaloid biosynthetic pathway candidate genes and random SNPs throughout the genome tested for significant differences in allele or genotype composition between the Wild Species and cultivated potato lines. Analysis was done using the diploid genotyping model. Bold numbers indicate significance at p-value≤0.05.

SNP Type	SNP	Chr	Position	PGSC Gene	Annotation	Allele Frequency p-value	Genotype Frequency p-value
Glycoalkaloid	solcap_snp_c1_15972	chr02	15346371	PGSC0003DMG400003461	3-hydroxy-3-methylglutaryl coenzyme A reductase (HMG2)	**0.0005**	**0.0000**
Glycoalkaloid	solcap_snp_c1_15975	chr02	15348125	PGSC0003DMG400003461	3-hydroxy-3-methylglutaryl coenzyme A reductase (HMG2)	**0.0043**	**0.0004**
Glycoalkaloid	solcap_snp_c1_9696	chr02	21888552	PGSC0003DMG400003324	squalene epoxidase (SQE)	**0.0000**	**0.0000**
Glycoalkaloid	solcap_snp_c2_32413	chr02	21888699	PGSC0003DMG400003324	squalene epoxidase (SQE)	**0.0007**	**0.0000**
Glycoalkaloid	solcap_snp_c2_32414	chr02	21889615	PGSC0003DMG400003324	squalene epoxidase (SQE)	0.1855	0.1691
Glycoalkaloid	solcap_snp_c2_32417	chr02	21892360	PGSC0003DMG400003324	squalene epoxidase (SQE)	**0.0000**	**0.0000**
Glycoalkaloid	solcap_snp_c2_23391	chr07	39727554	PGSC0003DMG400011749	UDP-galactose:solanidine galactosyltransferase (SGT1)	**0.0057**	**0.0008**
Glycoalkaloid	solcap_snp_c2_23390	chr07	39727884	PGSC0003DMG400011749	UDP-galactose:solanidine galactosyltransferase (SGT1)	0.1921	0.0894
Glycoalkaloid	solcap_snp_c2_23389	chr07	39727960	PGSC0003DMG400011749	UDP-galactose:solanidine galactosyltransferase (SGT1)	**0.0004**	0.0788
Glycoalkaloid	solcap_snp_c2_23388	chr07	39728034	PGSC0003DMG400011749	UDP-galactose:solanidine galactosyltransferase (SGT1)	0.2698	**0.0258**
Glycoalkaloid	solcap_snp_c2_23385	chr07	39728475	PGSC0003DMG400011749	UDP-galactose:solanidine galactosyltransferase (SGT1)	**0.0000**	**0.0000**
Glycoalkaloid	solcap_snp_c2_23384	chr07	39728673	PGSC0003DMG400011749	UDP-galactose:solanidine galactosyltransferase (SGT1)	0.3493	0.3404
Glycoalkaloid	solcap_snp_c2_23383	chr07	39728724	PGSC0003DMG400011749	UDP-galactose:solanidine galactosyltransferase (SGT1)	0.8660	**0.0007**
Glycoalkaloid	solcap_snp_c2_23382	chr07	39728727	PGSC0003DMG400011749	UDP-galactose:solanidine galactosyltransferase (SGT1)	0.9184	**0.0008**
Glycoalkaloid	solcap_snp_c2_23381	chr07	39728817	PGSC0003DMG400011749	UDP-galactose:solanidine galactosyltransferase (SGT1)	**0.0068**	**0.0000**
Glycoalkaloid	solcap_snp_c2_49600	chr08	35218101	PGSC0003DMG400017508	UDP-glucose:solanidine glucosyltransferase (SGT2)	**0.0010**	**0.0000**
Glycoalkaloid	solcap_snp_c2_49601	chr08	35218300	PGSC0003DMG400017508	UDP-glucose:solanidine glucosyltransferase (SGT2)	**0.0003**	**0.0000**
Glycoalkaloid	solcap_snp_c2_49602	chr08	35218570	PGSC0003DMG400017508	UDP-glucose:solanidine glucosyltransferase (SGT2)	**0.0001**	**0.0000**
Glycoalkaloid	solcap_snp_c2_49604	chr08	35219038	PGSC0003DMG400017508	UDP-glucose:solanidine glucosyltransferase (SGT2)	0.0732	**0.0420**
Random	solcap_snp_c2_20569	chr01	60048382	PGSC0003DMG400028985	Calcium ion binding protein	**0.0005**	**0.0000**
Random	solcap_snp_c2_49451	chr01	61451309	PGSC0003DMG400001685	Acyl carrier protein	**0.0011**	**0.0000**
Random	solcap_snp_c2_46455	chr01	78455375	PGSC0003DMG400025176	Non-symbiotic	**0.0142**	**0.0040**

					hemoglogin		
Random	solcap_snp_c2_55863	chr02	19615327	PGSC0003DMG400023897	Self-pruning G-box protein	**0.0000**	**0.0000**
Random	solcap_snp_c2_36264	chr03	582345	PGSC0003DMG400013391	Conserved gene of unknown function	**0.0016**	**0.0000**
Random	solcap_snp_c2_11479	chr04	5352996	PGSC0003DMG400027738	GTP-binding protein	0.3522	**0.0000**
Random	solcap_snp_c2_11758	chr05	1997209	PGSC0003DMG400000810	Serine-threonine protein kinase, plant-type	**0.0019**	**0.0000**
Random	solcap_snp_c2_9003	chr06	48341966	-	-	**0.0000**	**0.0000**
Random	solcap_snp_c2_35055	chr07	46414299	PGSC0003DMG400009380	Receptor protein kinase CLAVATA1	**0.0001**	**0.0000**
Random	solcap_snp_c2_19537	chr09	15802707	PGSC0003DMG400013943	Conserved gene of unknown function	**0.0000**	**0.0000**
Random	solcap_snp_c2_22003	chr09	45562360	PGSC0003DMG400032243	Homocysteine s-methyltransferase	0.1670	**0.0001**
Random	solcap_snp_c2_34	chr10	29455223	-	-	**0.0000**	**0.0000**
Random	solcap_snp_c1_4990	chr10	47875686	PGSC0003DMG400010454	Transitional endoplasmic reticulum ATPase	**0.0000**	**0.0000**
Random	solcap_snp_c2_23942	chr11	61738	PGSC0003DMG400000994	Calcium-dependent protein kinase 3	**0.0000**	**0.0000**
Random	solcap_snp_c2_15342	chr11	38767963	PGSC0003DMG400008081	Auxin response factor	0.0734	**0.0000**
Random	solcap_snp_c2_15332	chr11	38770184	PGSC0003DMG400008081	Auxin response factor	**0.0010**	**0.0000**
Random	solcap_snp_c1_15926	UM	UM	PGSC0003DMG400022947	Uncharacterized aarF domain-containing protein kinase, chloroplastic	**0.0045**	**0.0007**

Table S8 Significance of model effects for the 190 tetraploid lines with phenotypic data. Three replications of the lines were evaluated in 2010 (two replications at the Wisconsin location and one replication at the New York location).

Source of Variation	Snack Food Association Chip Color	Tuber Glucose Concentration	Tuber Sucrose Concentration	Tuber Shape
Entry	**	**	**	**
Replication	*	*	**	NS

* = significant at 0.01; ** = significant at 0.001; NS = not significant

a. Spearman rank correlations between all replicates were significant at p=0.05

b. Spearman rank correlations between replicates 1 and 2 and between 1 and 3 were significant and between replicates 2 and 3 were not significant at p=0.05

Table S9 Least square means of 190 tetraploid lines with phenotypic data. Three replications of the lines were evaluated in 2010 (two replications at the Wisconsin location and one replication at the New York Location).

Clone	Snack Food Association Chip Color	Tuber Glucose Concentration	Tuber Sucrose Concentration	Tuber Shape
A96104-2	2.833	2.340	7.760	4.667
A97066-42	2.167	0.480	9.840	4.667
AC Brador	3.000	2.183	5.367	3.667
Adirondack Blue	3.167	3.870	6.387	3.667
Adirondack Red	2.667	1.257	5.353	4.000
AF2291-10	1.500	0.310	6.367	2.667
AF2376-5	1.833	0.533	6.650	2.667
AF2574-1	3.333	1.597	5.463	3.333
All Blue	2.500	0.817	7.233	5.000
Alpine Russet	3.167	1.493	9.113	4.667
Alturas	2.333	1.060	6.273	4.333
Andover	2.167	0.757	6.040	2.667
AO96141-3	2.667	1.113	8.090	5.000
AO96160-3	2.833	1.023	6.160	5.000
AO96164-1	2.500	0.827	6.757	5.000
AWN86514-2	1.833	0.683	8.703	4.333
B1829-5	1.833	0.220	5.297	2.667
Bannock Russet	2.667	0.360	6.417	4.667
Beacon Chipper	2.000	0.160	5.677	2.667
Bintje	2.667	1.260	5.890	4.000
Blazer Russet	3.000	1.270	7.280	5.000
Boulder	1.667	0.160	4.023	2.000
C31-5-115	3.167	2.563	8.763	2.000
C31-5-120	2.667	0.827	6.873	2.000
C5	1.667	0.473	8.123	3.333
Canela Russet	2.667	2.703	8.247	5.000
Cascade	4.000	4.020	8.090	4.333
Centennial Russet	4.167	5.363	15.633	3.667
Chieftain	3.833	4.720	7.533	3.333
Chipeta	1.833	0.347	4.080	3.333
Chippewa	3.000	1.957	6.523	3.333
Classic Russet	3.500	3.750	7.407	5.000
Clearwater Russet	1.500	0.183	4.870	4.667
CV96022-3	1.667	0.563	4.753	4.333

CV98112-3	2.667	0.860	7.123	5.000
Dakota Crisp	1.167	0.197	5.353	2.667
Dakota Diamond	1.833	0.170	6.217	2.667
Dakota Jewel	3.000	4.280	9.257	2.667
Dakota Pearl	1.500	0.570	4.727	2.667
Dark Red Norland	4.000	7.187	12.350	3.333
Defender	2.833	1.690	7.960	4.333
Denali	2.167	0.807	6.137	2.667
Early Rose	3.333	1.637	6.383	4.667
Elba	3.667	3.673	8.540	2.333
Eramosa	3.833	5.320	5.463	2.667
Eva	1.833	1.079	5.512	2.667
Exploits	3.167	1.310	7.577	2.667
F02018	2.167	0.643	4.760	3.333
F58050	1.067	NA	6.657	1.994
F66041	1.567	NA	6.267	4.994
F87084	2.500	2.433	4.263	2.667
Freedom Russet	3.000	1.547	4.770	5.000
Garnet Chile	3.217	2.139	10.027	4.003
GemStar Russet	2.167	0.710	5.850	4.667
Grand Falls	2.167	0.490	6.963	4.000
Green Mountain	3.667	2.213	10.323	4.333
Highland Russet	3.000	0.857	6.517	4.667
Hindenburg	2.500	0.837	6.323	3.667
Ida Rose	4.000	5.483	9.637	2.667
Inca Gold	2.667	1.767	12.583	1.667
Irish Cobbler	3.250	3.393	8.170	3.000
Ivory Crisp	1.333	0.163	3.593	2.000
Jacqueline Lee	2.667	0.973	9.467	3.667
Kalkaska	1.667	0.297	5.600	1.667
Katahdin	3.333	5.103	5.920	3.333
Kennebec	2.500	1.150	4.007	4.000
Keuka Gold	2.167	0.743	4.480	2.667
King Harry (NY131)	2.167	1.337	5.613	2.000
Klamath Russet	2.667	0.617	7.823	4.667
La Chipper	2.833	2.107	5.790	2.667
Lamoka (NY139)	1.167	0.117	5.590	2.667
Langlade	2.500	0.687	5.060	2.667
Lehigh	2.667	0.890	5.007	2.667

Lelah (W2717-5)	1.000	0.250	4.663	2.667
Liberator	1.333	0.470	6.613	2.667
MaineChip	1.167	0.210	7.330	2.000
Marcy	1.167	0.177	3.313	3.333
MegaChip (W1201)	1.667	0.193	4.487	2.667
Michigan Purple	3.500	3.710	6.953	2.667
Missaukee (MSJ461-1)	1.500	0.263	5.360	1.667
MN 02419	2.167	0.583	15.583	2.000
MN 02467	3.000	1.530	3.843	5.000
MN 02586	1.667	0.207	8.753	1.333
MN 15620	2.500	1.213	9.493	4.333
MN 18747	2.833	3.727	5.990	4.333
MN 19298	3.833	4.017	9.097	2.000
MN 19350	2.833	1.690	6.827	3.333
MN 19470	1.833	0.917	6.447	2.667
MN 96072-4	2.667	0.827	9.957	3.333
MN 99380-1	1.833	0.480	7.403	2.333
Modoc	3.167	1.940	5.510	2.667
Monona	2.167	0.293	6.030	2.667
Monticello	1.583	0.337	4.720	2.333
Mountain Rose	2.667	2.237	6.667	3.333
MSG227-2	1.333	0.120	3.727	3.333
MSH228-6	1.167	0.237	6.993	2.333
MSI005-20Y	2.833	1.620	8.163	2.333
MSJ126-9Y	1.067	NA	3.987	1.994
MSJ147-1	1.000	0.150	8.507	3.333
MSK061-4	1.333	0.517	5.307	2.667
MSK409-1	1.000	0.133	5.267	2.667
MSL211-3	4.000	7.073	8.430	3.333
MSL268-D	1.500	0.200	6.050	2.667
MSL292-A	1.000	0.127	5.123	1.000
MSL512-6	1.333	0.233	7.400	3.003
MSL766-1	2.000	0.340	8.550	2.000
MSM051-3	1.333	0.200	5.253	2.667
MSM171-A	4.333	13.907	7.997	3.333
MSM182-1	2.667	2.227	4.913	2.333
MSM246-B	1.167	0.110	5.400	2.000
MSN105-1	3.167	1.757	9.173	2.333
MSN191-2Y	1.000	0.277	3.977	2.667

MSN215-2P	3.667	5.450	12.510	3.000
MSP239-1	1.833	0.700	5.590	2.000
MSQ070-1	1.167	0.073	5.260	2.000
MSQ176-5	2.333	0.177	5.003	2.000
MSQ279-1	1.667	0.290	4.340	2.000
MSR061-1	1.333	0.177	4.117	2.000
MSR160-2Y	1.333	0.240	8.127	2.000
MWTX 2609-2RU	3.167	1.957	5.593	5.000
ND1215-1 (NDSU 6)	2.167	0.460	5.603	3.333
ND2858-1 (NDSU 4)	2.167	1.070	13.413	3.333
ND8229-3 (NDSU 5)	1.500	0.327	5.427	5.000
ND8555-8R (NDSU 3)	3.167	2.607	9.240	3.333
ND860-2	1.500	0.137	5.563	2.000
Nicolet (W2133-1)	1.167	0.103	3.257	1.667
Norchip	2.333	0.230	8.073	2.667
NorDonna	4.000	1.620	6.320	2.333
Northstar	2.333	0.793	5.330	3.333
NorValley	1.333	0.510	4.370	2.333
Norwis (FL 657)	1.833	0.463	5.430	2.667
NY115	1.167	0.173	5.927	2.667
NY121	2.333	0.623	7.153	1.333
NY136	2.833	0.420	6.907	3.333
NY140	2.167	0.943	5.750	3.333
NY142	4.167	5.093	11.823	2.333
NYE48-2	1.000	0.167	3.697	3.333
Ontario	3.333	3.160	7.023	2.667
OR00068-11	2.500	1.013	9.103	2.667
PA99N82-4	2.967	1.134	6.492	3.503
Patagonia (NDSU 2)	3.667	2.203	7.567	4.333
Peter Wilcox	2.000	0.720	5.787	3.333
Pike	1.167	0.100	3.633	2.333
POR01PG1-6	NA	0.613	6.297	4.994
POR01PG16-1	1.833	1.173	7.507	5.000
POR01PG22-1	2.500	0.794	4.637	5.000
POR02PG37-2	2.667	1.170	7.903	2.667
Premier Russet	1.833	0.167	8.263	4.333
Prince Hairy (NY235-4)	2.667	1.757	6.700	2.000
Purple Majesty	1.667	0.287	6.290	3.333
Purple Peruvian	1.667	2.313	9.317	5.000

Ranger Russet	3.000	0.577	8.363	5.000
Reba	1.333	0.317	4.823	3.333
Red Maria (NY129)	3.167	1.780	7.243	2.333
Reeves Kingpin	3.167	3.233	6.520	5.000
Rideau	3.167	1.263	8.643	2.667
Rochdale Gold- Doree	3.000	1.460	7.487	2.000
Rosa	2.167	0.430	6.807	2.667
Russet Burbank	2.667	0.980	6.937	4.667
Russet Norkotah-S3	3.167	2.233	8.163	4.333
Russet Norkotah-S8	3.500	2.160	6.167	4.333
Salem	3.167	2.653	9.317	3.333
Satina	3.500	3.177	6.960	2.667
Sebago	3.000	4.703	6.027	2.667
Sierra Gold	2.667	0.517	6.420	3.333
Silverton Russet	2.500	0.797	5.013	5.000
Snowden	1.167	0.127	4.277	2.000
Superior	2.833	2.387	6.607	2.667
TrailBlazer Russet (ROND95249-Russ)	1.833	0.340	5.147	4.333
Tundra (W2310-3)	1.000	0.120	4.357	2.333
Umatilla Russet	2.500	2.497	6.087	5.000
V1102-1	3.667	2.373	10.907	4.333
V1115-3	2.667	1.117	5.257	4.333
Villetta Rose	3.167	1.300	5.230	2.667
W1151rus	3.333	4.800	7.500	5.000
W2253-5rus	3.500	2.907	8.383	4.333
W2309-7	1.000	0.153	4.087	3.333
W2683-2rus	2.333	0.653	7.763	5.000
W4013-1	1.167	0.137	4.877	2.333
Wallowa Russet	2.667	0.523	6.663	5.000
Waneta (NY138)	1.167	0.140	3.870	3.333
Western russet	3.000	1.030	6.427	4.333
White Pearl	1.000	0.101	3.158	2.500
Willamette	1.067	NA	4.107	NA
Winema	3.167	2.307	6.123	3.333
Yankee Chipper	2.000	0.287	5.457	3.667
Yukon Gem	1.833	0.603	5.227	3.333
Yukon Gold	3.333	1.210	7.190	3.000
Rio Grande Russet	3.000	1.373	7.333	5.000
Atlantic	1.333	0.143	4.660	2.000

Table S10 Summary of single nucleotide polymorphisms (SNPs) in carotenoid biosynthetic pathway genes tested for significant differences in allele or genotype composition compared to that observed in all other cultivated potato lines. SNPs with a p-value≤0.05 were considered significant.

PGSC Gene	Annotation	Significant Allele Frequency (Significant SNPs / Total SNPs)	Significant Genotype Frequency (Significant SNPs / Total SNPs)
PGSC0003DMG400013335	pTOX - alternative oxidase	0/1	0/1
PGSC0003DMG400028897	CHY1 - beta-carotene hydroxylase	1/1	1/1
PGSC0003DMG400008159	LCY-b - lycopene beta cyclase, chloroplastic	0/3	0/3
PGSC0003DMG400010637	LCY-b - lycopene beta cyclase, chloroplastic	0/2	1/2
PGSC0003DMG400009156	PDS - phytoene dehydrogenase, chloroplastic/chromoplastic	0/2	1/2
PGSC0003DMG400028224	CrtISO - carotenoid isomerase, chloroplastic	0/2	0/2
PGSC0003DMG400022473	ZDS - zeta-carotene desaturase	2/4	1/4
PGSC0003DMG400000333	LCY-e - lycopene epsilon cyclase	0/3	1/3
