# Supplementary figures and images for: Retrospective View of North American Potato (Solanum tuberosum L.) Breeding in the 20th and 21st Centuries
Source: G3 (Bethesda). 2013 Jun 1;3(6):1003–13. doi: 10.1534/g3.113.005595 (PMC3689798; doi:10.1534/g3.113.005595)

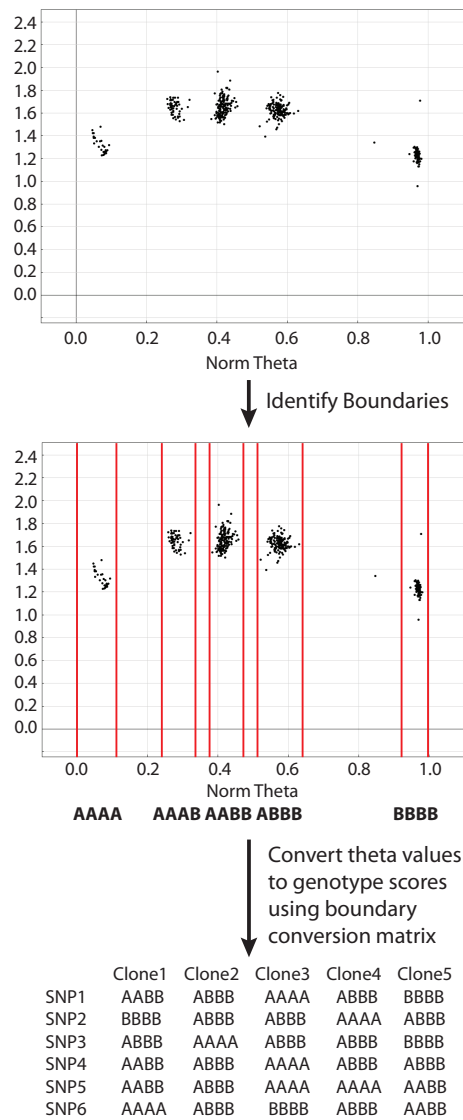

**Figure S2** Workflow used to determine dosage genotype scores.

Supplement: Supporting Information [file supp_g3.113.005595_FigureS2.pdf]

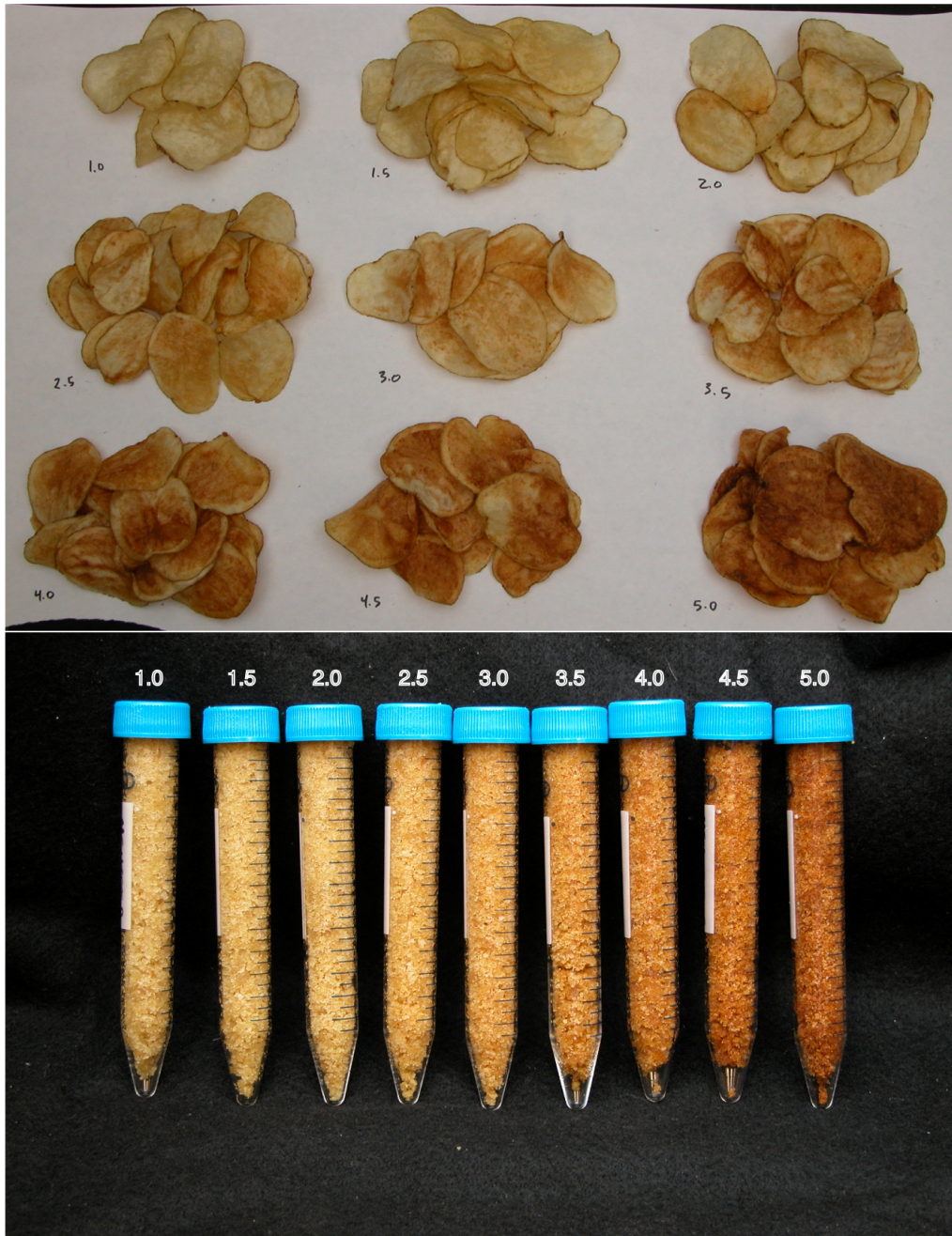

**Figure S3** Snack Food Association (SFA) chip color measurement standard.

Supplement: Supporting Information [file supp_g3.113.005595_FigureS3.pdf]

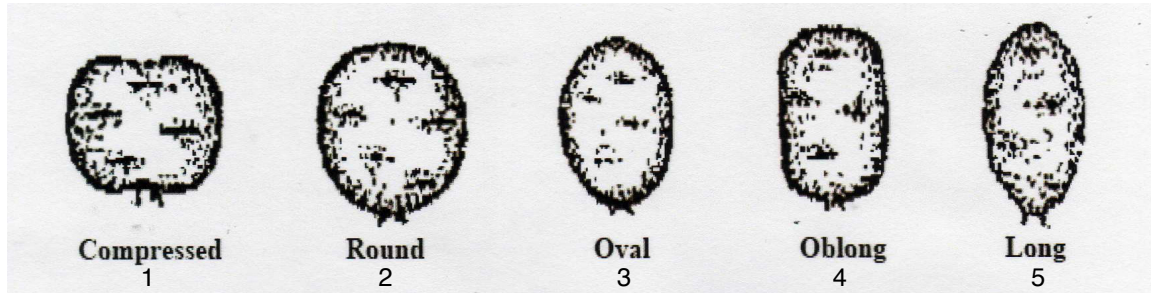

**Figure S4** Tuber shape measurement standard.

Supplement: Supporting Information [file supp_g3.113.005595_FigureS4.pdf]
