# Supplementary material for: Retrospective View of North American Potato (Solanum tuberosum L.) Breeding in the 20th and 21st Centuries
Source: G3 (Bethesda). 2013 Jun 1;3(6):1003–13. doi: 10.1534/g3.113.005595 (PMC3689798; doi:10.1534/g3.113.005595)
Supplement: Supporting Information [file supp_g3.113.005595_FigureS5.pdf]

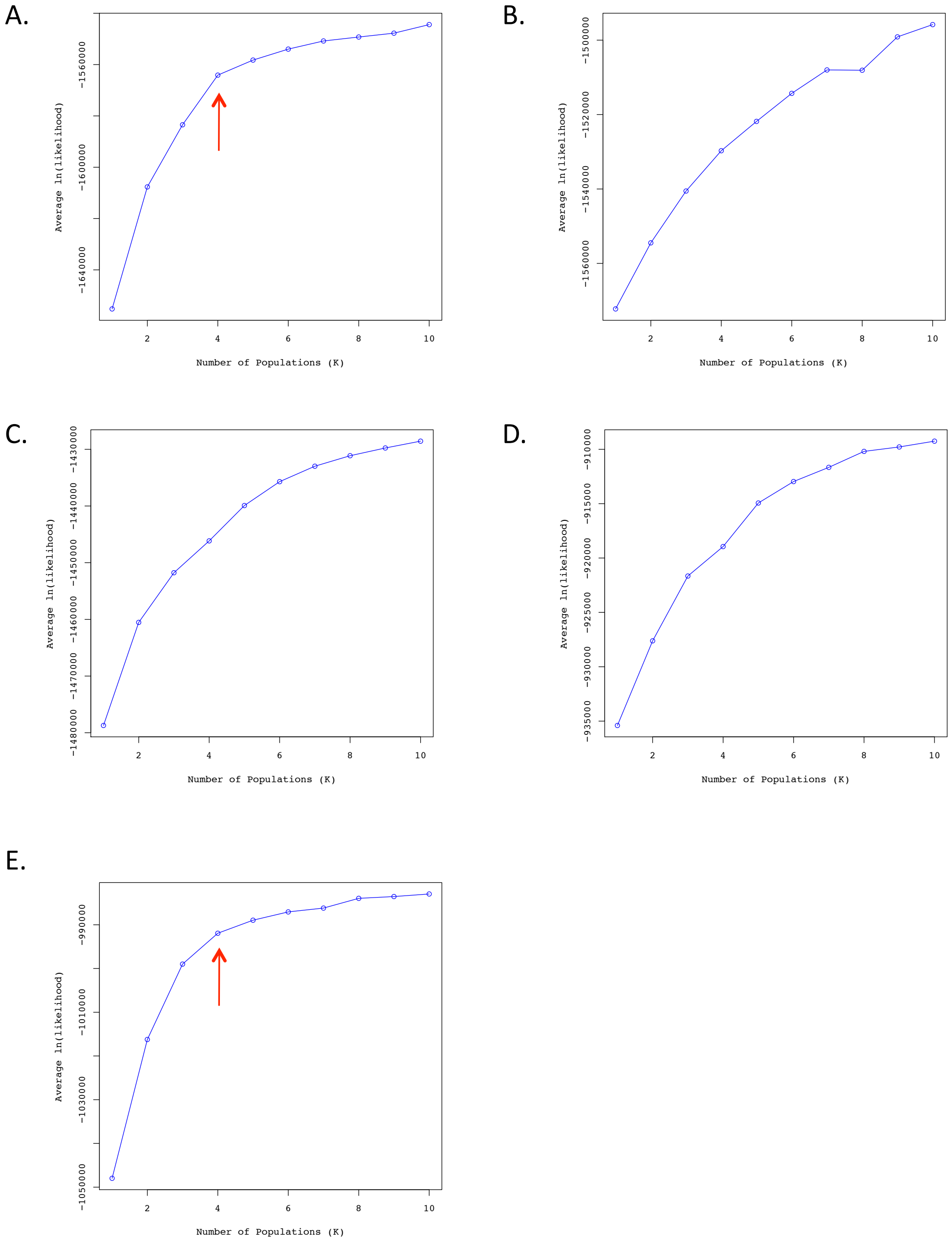

Figure S5 Average likelihood estimates from STRUCTURE with varying *K* (number of populations) values. STRUCTURE was run on multiple datasets, and likelihood estimates are shown for (A) diploid dataset, (B) tetraploid as tetraploid dataset, (C) tetraploid as diploid dataset, (D) tetraploid as diploid subset, and (E) diploid subset. The red arrows indicate the inflection points in each graph if a clear inflection point was observed.
