# Supplementary material for: Retrospective View of North American Potato (Solanum tuberosum L.) Breeding in the 20th and 21st Centuries
Source: G3 (Bethesda). 2013 Jun 1;3(6):1003–13. doi: 10.1534/g3.113.005595 (PMC3689798; doi:10.1534/g3.113.005595)
Supplement: Supporting Information [file supp_g3.113.005595_FigureS6.pdf]

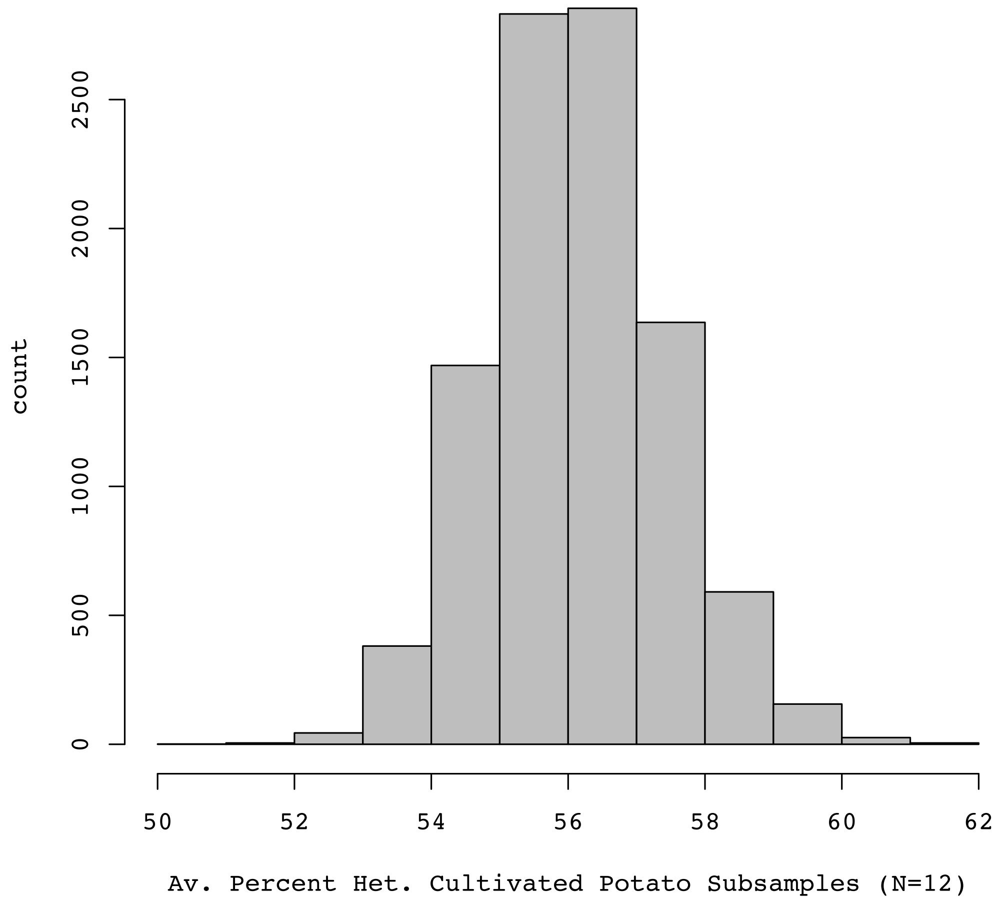


**Figure S6** Histogram of average percent heterozygosity in subsamples (N=12) of the cultivated potato clones. Sub-sampling was performed with replacement 10,000 times.
