# Supplementary material for: Retrospective View of North American Potato (Solanum tuberosum L.) Breeding in the 20th and 21st Centuries
Source: G3 (Bethesda). 2013 Jun 1;3(6):1003–13. doi: 10.1534/g3.113.005595 (PMC3689798; doi:10.1534/g3.113.005595)
Supplement: Supporting Information [file supp_g3.113.005595_FileS1.pdf]

File S1 Summary of STRUCTURE validation testing the effects of varying marker number, genotype method, and germplasm included in the analysis.

STRUCTURE (PRITCHARD *et al.* 2000) analysis was conducted with only the tetraploid clones (221 clones) using the dosage genotyping model (AAAA, AAAB, AABB, ABBB, BBBB; 3,763 markers) to evaluate if subpopulations exist within cultivated potato that could not be resolved due to the inclusion of Genetic Stock and Wild Species in the previous analysis. However, no inflection point was observed in the maximum likelihood graphs with a maximum *K* value of 10 (Figure S5B). In addition to removing the Genetic Stock and Wild Species, this analysis used a subset of the markers and dosage genotyping calls. To confirm that the lack of observed structure was not from either of these variables, analysis of the tetraploid clones was repeated using the diploid genotyping model (AA, AB, BB) with all of the markers that could be scored (6,373 markers; Figure S5C) and with the subset of markers that dosage genotyping could be called on (3,763 markers; Figure S5D) and again no inflection point was observed. However, repeating the analysis with the entire panel (250 clones) using the diploid genotyping model but only with the subset of markers that dosage genotyping could be called on (3,763 markers) a clear inflection point was again observed for *K*=4 (Figure S5E).

PRITCHARD, J. K., M. STEPHENS and P. DONNELLY, 2000 Inference of population structure using multilocus genotype data. Genetics **155:** 945-959.
