# Supplementary material for: Retrospective View of North American Potato (Solanum tuberosum L.) Breeding in the 20th and 21st Centuries
Source: G3 (Bethesda). 2013 Jun 1;3(6):1003–13. doi: 10.1534/g3.113.005595 (PMC3689798; doi:10.1534/g3.113.005595)
Supplement: Supporting Information [file supp_g3.113.005595_TableS5.pdf]

Table S5 Single nucleotide polymorphisms (SNPs) in carotenoid biosynthetic pathway genes and random SNPs tested for significant differences in allele or genotype composition compared to that observed in all other cultivated potato lines. Bold numbers indicate significance at p-value≤0.05.

SNP Type	SNP Name	PGSC Gene	Annotation	Allele Frequency p-value	Genotype Frequency p-value
Carotenoid	solcap_snp_c2_13431	PGSC0003DMG400013335	alternative oxidase	0.503	0.622
Carotenoid	solcap_snp_c1_12691	PGSC0003DMG400028897	beta-carotene hydroxylase	**0.006**	**0.005**
Carotenoid	solcap_snp_c2_45240	PGSC0003DMG400008159	lycopene beta cyclase, chloroplastic	0.976	0.886
Carotenoid	solcap_snp_c2_45242	PGSC0003DMG400008159	lycopene beta cyclase, chloroplastic	0.917	0.648
Carotenoid	solcap_snp_c2_45244	PGSC0003DMG400008159	lycopene beta cyclase, chloroplastic	0.875	0.668
Carotenoid	solcap_snp_c2_51559	PGSC0003DMG400010637	lycopene beta cyclase, chloroplastic	0.802	0.222
Carotenoid	solcap_snp_c2_51560	PGSC0003DMG400010637	lycopene beta cyclase, chloroplastic	0.220	**0.045**
Carotenoid	solcap_snp_c2_37118	PGSC0003DMG400009156	phytoene dehydrogenase, chloroplastic/chromoplastic	0.345	0.195
Carotenoid	solcap_snp_c2_37121	PGSC0003DMG400009156	phytoene dehydrogenase, chloroplastic/chromoplastic	0.070	**0.035**
Carotenoid	solcap_snp_c1_9060	PGSC0003DMG400028224	carotenoid isomerase, chloroplastic	0.131	0.417
Carotenoid	solcap_snp_c2_29741	PGSC0003DMG400028224	carotenoid isomerase, chloroplastic	0.589	0.051
Carotenoid	solcap_snp_c1_5286	PGSC0003DMG400022473	zeta-carotene desaturase	**0.010**	0.239
Carotenoid	solcap_snp_c2_16424	PGSC0003DMG400022473	zeta-carotene desaturase	0.236	0.070
Carotenoid	solcap_snp_c2_16425	PGSC0003DMG400022473	zeta-carotene desaturase	0.217	**0.011**
Carotenoid	solcap_snp_c2_16426	PGSC0003DMG400022473	zeta-carotene desaturase	**0.019**	0.300
Carotenoid	solcap_snp_c2_27926	PGSC0003DMG400000333	lycopene epsilon cyclase	0.687	0.723
Carotenoid	solcap_snp_c2_27929	PGSC0003DMG400000333	lycopene epsilon cyclase	0.438	**0.031**
Carotenoid	solcap_snp_c2_27930	PGSC0003DMG400000333	lycopene epsilon cyclase	0.512	0.363
Random	solcap_snp_c1_13189	PGSC0003DMG400022953	pyruvate decarboxylase	0.370	0.360
Random	solcap_snp_c1_4990	PGSC0003DMG400010454	transitional endoplasmic reticulum ATPase	0.356	0.123
Random	solcap_snp_c2_11479	PGSC0003DMG400027738	GTP-binding protein	0.829	0.384
Random	solcap_snp_c2_11758	PGSC0003DMG400000810	serine-threonine protein kinase, plant-type	0.604	0.524
Random	solcap_snp_c2_15332	PGSC0003DMG400008081	auxin response factor	0.171	0.326
Random	solcap_snp_c2_15342	PGSC0003DMG400008081	auxin response factor	0.122	0.313
Random	solcap_snp_c2_19537	PGSC0003DMG400013943	conserved gene of unknown function	0.194	**0.032**
Random	solcap_snp_c2_22003	PGSC0003DMG400032243	homocysteine s-methyltransferase	**0.017**	0.065
Random	solcap_snp_c2_23942	PGSC0003DMG400000994	calcium-dependent protein kinase 3	0.925	0.222
Random	solcap_snp_c2_34	-	-	0.527	0.740
Random	solcap_snp_c2_35055	PGSC0003DMG400009380	receptor protein kinase CLAVATA1	**0.017**	**0.020**
Random	solcap_snp_c2_36264	PGSC0003DMG400013391	conserved gene of unknown function	0.721	0.792
Random	solcap_snp_c2_46455	PGSC0003DMG400025176	non-symbiotic hemoglogin	0.908	0.474
Random	solcap_snp_c2_55863	PGSC0003DMG400023897	self-pruning G-box protein	0.079	0.562
Random	solcap_snp_c2_9003	-	-	0.267	0.847
Random	solcap_snp_c1_15926	PGSC0003DMG400022947	uncharacterized aarF domain-containing protein kinase, chloroplastic	**0.003**	0.062
Random	solcap_snp_c2_20569	PGSC0003DMG400028985	calcium ion binding protein	0.849	0.262
Random	solcap_snp_c2_49451	PGSC0003DMG400001685	acyl carrier protein	0.252	0.296
