# Supplementary material for: Retrospective View of North American Potato (Solanum tuberosum L.) Breeding in the 20th and 21st Centuries
Source: G3 (Bethesda). 2013 Jun 1;3(6):1003–13. doi: 10.1534/g3.113.005595 (PMC3689798; doi:10.1534/g3.113.005595)
Supplement: Supporting Information [file supp_g3.113.005595_TableS7.pdf]

Table S7 Single nucleotide polymorphisms (SNPs) in glycoalkaloid biosynthetic pathway candidate genes and random SNPs throughout the genome tested for significant differences in allele or genotype composition between the Wild Species and cultivated potato lines. Analysis was done using the diploid genotyping model. Bold numbers indicate significance at p-value≤0.05.

SNP Type	SNP	Chr	Position	PGSC Gene	Annotation	Allele Frequency p-value	Genotype Frequency p-value
Glycoalkaloid	solcap_snp_c1_15972	chr02	15346371	PGSC0003DMG400003461	3-hydroxy-3-methylglutaryl coenzyme A reductase (HMG2)	**0.0005**	**0.0000**
Glycoalkaloid	solcap_snp_c1_15975	chr02	15348125	PGSC0003DMG400003461	3-hydroxy-3-methylglutaryl coenzyme A reductase (HMG2)	**0.0043**	**0.0004**
Glycoalkaloid	solcap_snp_c1_9696	chr02	21888552	PGSC0003DMG400003324	squalene epoxidase (SQE)	**0.0000**	**0.0000**
Glycoalkaloid	solcap_snp_c2_32413	chr02	21888699	PGSC0003DMG400003324	squalene epoxidase (SQE)	**0.0007**	**0.0000**
Glycoalkaloid	solcap_snp_c2_32414	chr02	21889615	PGSC0003DMG400003324	squalene epoxidase (SQE)	0.1855	0.1691
Glycoalkaloid	solcap_snp_c2_32417	chr02	21892360	PGSC0003DMG400003324	squalene epoxidase (SQE)	**0.0000**	**0.0000**
Glycoalkaloid	solcap_snp_c2_23391	chr07	39727554	PGSC0003DMG400011749	UDP-galactose:solanidine galactosyltransferase (SGT1)	**0.0057**	**0.0008**
Glycoalkaloid	solcap_snp_c2_23390	chr07	39727884	PGSC0003DMG400011749	UDP-galactose:solanidine galactosyltransferase (SGT1)	0.1921	0.0894
Glycoalkaloid	solcap_snp_c2_23389	chr07	39727960	PGSC0003DMG400011749	UDP-galactose:solanidine galactosyltransferase (SGT1)	**0.0004**	0.0788
Glycoalkaloid	solcap_snp_c2_23388	chr07	39728034	PGSC0003DMG400011749	UDP-galactose:solanidine galactosyltransferase (SGT1)	0.2698	**0.0258**
Glycoalkaloid	solcap_snp_c2_23385	chr07	39728475	PGSC0003DMG400011749	UDP-galactose:solanidine galactosyltransferase (SGT1)	**0.0000**	**0.0000**
Glycoalkaloid	solcap_snp_c2_23384	chr07	39728673	PGSC0003DMG400011749	UDP-galactose:solanidine galactosyltransferase (SGT1)	0.3493	0.3404
Glycoalkaloid	solcap_snp_c2_23383	chr07	39728724	PGSC0003DMG400011749	UDP-galactose:solanidine galactosyltransferase (SGT1)	0.8660	**0.0007**
Glycoalkaloid	solcap_snp_c2_23382	chr07	39728727	PGSC0003DMG400011749	UDP-galactose:solanidine galactosyltransferase (SGT1)	0.9184	**0.0008**
Glycoalkaloid	solcap_snp_c2_23381	chr07	39728817	PGSC0003DMG400011749	UDP-galactose:solanidine galactosyltransferase (SGT1)	**0.0068**	**0.0000**
Glycoalkaloid	solcap_snp_c2_49600	chr08	35218101	PGSC0003DMG400017508	UDP-glucose:solanidine glucosyltransferase (SGT2)	**0.0010**	**0.0000**
Glycoalkaloid	solcap_snp_c2_49601	chr08	35218300	PGSC0003DMG400017508	UDP-glucose:solanidine glucosyltransferase (SGT2)	**0.0003**	**0.0000**
Glycoalkaloid	solcap_snp_c2_49602	chr08	35218570	PGSC0003DMG400017508	UDP-glucose:solanidine glucosyltransferase (SGT2)	**0.0001**	**0.0000**
Glycoalkaloid	solcap_snp_c2_49604	chr08	35219038	PGSC0003DMG400017508	UDP-glucose:solanidine glucosyltransferase (SGT2)	0.0732	**0.0420**
Random	solcap_snp_c2_20569	chr01	60048382	PGSC0003DMG400028985	Calcium ion binding protein	**0.0005**	**0.0000**
Random	solcap_snp_c2_49451	chr01	61451309	PGSC0003DMG400001685	Acyl carrier protein	**0.0011**	**0.0000**
Random	solcap_snp_c2_46455	chr01	78455375	PGSC0003DMG400025176	Non-symbiotic	**0.0142**	**0.0040**

					hemoglogin		
Random	solcap_snp_c2_55863	chr02	19615327	PGSC0003DMG400023897	Self-pruning G-box protein	**0.0000**	**0.0000**
Random	solcap_snp_c2_36264	chr03	582345	PGSC0003DMG400013391	Conserved gene of unknown function	**0.0016**	**0.0000**
Random	solcap_snp_c2_11479	chr04	5352996	PGSC0003DMG400027738	GTP-binding protein	0.3522	**0.0000**
Random	solcap_snp_c2_11758	chr05	1997209	PGSC0003DMG400000810	Serine-threonine protein kinase, plant-type	**0.0019**	**0.0000**
Random	solcap_snp_c2_9003	chr06	48341966	-	-	**0.0000**	**0.0000**
Random	solcap_snp_c2_35055	chr07	46414299	PGSC0003DMG400009380	Receptor protein kinase CLAVATA1	**0.0001**	**0.0000**
Random	solcap_snp_c2_19537	chr09	15802707	PGSC0003DMG400013943	Conserved gene of unknown function	**0.0000**	**0.0000**
Random	solcap_snp_c2_22003	chr09	45562360	PGSC0003DMG400032243	Homocysteine s-methyltransferase	0.1670	**0.0001**
Random	solcap_snp_c2_34	chr10	29455223	-	-	**0.0000**	**0.0000**
Random	solcap_snp_c1_4990	chr10	47875686	PGSC0003DMG400010454	Transitional endoplasmic reticulum ATPase	**0.0000**	**0.0000**
Random	solcap_snp_c2_23942	chr11	61738	PGSC0003DMG400000994	Calcium-dependent protein kinase 3	**0.0000**	**0.0000**
Random	solcap_snp_c2_15342	chr11	38767963	PGSC0003DMG400008081	Auxin response factor	0.0734	**0.0000**
Random	solcap_snp_c2_15332	chr11	38770184	PGSC0003DMG400008081	Auxin response factor	**0.0010**	**0.0000**
Random	solcap_snp_c1_15926	UM	UM	PGSC0003DMG400022947	Uncharacterized aarF domain-containing protein kinase, chloroplastic	**0.0045**	**0.0007**
