# Supplementary material for: Retrospective View of North American Potato (Solanum tuberosum L.) Breeding in the 20th and 21st Centuries
Source: G3 (Bethesda). 2013 Jun 1;3(6):1003–13. doi: 10.1534/g3.113.005595 (PMC3689798; doi:10.1534/g3.113.005595)
Supplement: Supporting Information [file supp_g3.113.005595_TableS8.pdf]

Table S8 Significance of model effects for the 190 tetraploid lines with phenotypic data. Three replications of the lines were evaluated in 2010 (two replications at the Wisconsin location and one replication at the New York location).

Source of Variation	Snack Food Association Chip Color	Tuber Glucose Concentration	Tuber Sucrose Concentration	Tuber Shape
Entry	**	**	**	**
Replication	*	*	**	NS

* = significant at 0.01; ** = significant at 0.001; NS = not significant

a. Spearman rank correlations between all replicates were significant at p=0.05

b. Spearman rank correlations between replicates 1 and 2 and between 1 and 3 were significant and between replicates 2 and 3 were not significant at p=0.05
