# Supplementary material for: Retrospective View of North American Potato (Solanum tuberosum L.) Breeding in the 20th and 21st Centuries
Source: G3 (Bethesda). 2013 Jun 1;3(6):1003–13. doi: 10.1534/g3.113.005595 (PMC3689798; doi:10.1534/g3.113.005595)
Supplement: Supporting Information [file supp_g3.113.005595_TableS9.pdf]

Table S9 Least square means of 190 tetraploid lines with phenotypic data. Three replications of the lines were evaluated in 2010 (two replications at the Wisconsin location and one replication at the New York Location).

Clone	Snack Food Association Chip Color	Tuber Glucose Concentration	Tuber Sucrose Concentration	Tuber Shape
A96104-2	2.833	2.340	7.760	4.667
A97066-42	2.167	0.480	9.840	4.667
AC Brador	3.000	2.183	5.367	3.667
Adirondack Blue	3.167	3.870	6.387	3.667
Adirondack Red	2.667	1.257	5.353	4.000
AF2291-10	1.500	0.310	6.367	2.667
AF2376-5	1.833	0.533	6.650	2.667
AF2574-1	3.333	1.597	5.463	3.333
All Blue	2.500	0.817	7.233	5.000
Alpine Russet	3.167	1.493	9.113	4.667
Alturas	2.333	1.060	6.273	4.333
Andover	2.167	0.757	6.040	2.667
AO96141-3	2.667	1.113	8.090	5.000
AO96160-3	2.833	1.023	6.160	5.000
AO96164-1	2.500	0.827	6.757	5.000
AWN86514-2	1.833	0.683	8.703	4.333
B1829-5	1.833	0.220	5.297	2.667
Bannock Russet	2.667	0.360	6.417	4.667
Beacon Chipper	2.000	0.160	5.677	2.667
Bintje	2.667	1.260	5.890	4.000
Blazer Russet	3.000	1.270	7.280	5.000
Boulder	1.667	0.160	4.023	2.000
C31-5-115	3.167	2.563	8.763	2.000
C31-5-120	2.667	0.827	6.873	2.000
C5	1.667	0.473	8.123	3.333
Canela Russet	2.667	2.703	8.247	5.000
Cascade	4.000	4.020	8.090	4.333
Centennial Russet	4.167	5.363	15.633	3.667
Chieftain	3.833	4.720	7.533	3.333
Chipeta	1.833	0.347	4.080	3.333
Chippewa	3.000	1.957	6.523	3.333
Classic Russet	3.500	3.750	7.407	5.000
Clearwater Russet	1.500	0.183	4.870	4.667
CV96022-3	1.667	0.563	4.753	4.333

CV98112-3	2.667	0.860	7.123	5.000
Dakota Crisp	1.167	0.197	5.353	2.667
Dakota Diamond	1.833	0.170	6.217	2.667
Dakota Jewel	3.000	4.280	9.257	2.667
Dakota Pearl	1.500	0.570	4.727	2.667
Dark Red Norland	4.000	7.187	12.350	3.333
Defender	2.833	1.690	7.960	4.333
Denali	2.167	0.807	6.137	2.667
Early Rose	3.333	1.637	6.383	4.667
Elba	3.667	3.673	8.540	2.333
Eramosa	3.833	5.320	5.463	2.667
Eva	1.833	1.079	5.512	2.667
Exploits	3.167	1.310	7.577	2.667
F02018	2.167	0.643	4.760	3.333
F58050	1.067	NA	6.657	1.994
F66041	1.567	NA	6.267	4.994
F87084	2.500	2.433	4.263	2.667
Freedom Russet	3.000	1.547	4.770	5.000
Garnet Chile	3.217	2.139	10.027	4.003
GemStar Russet	2.167	0.710	5.850	4.667
Grand Falls	2.167	0.490	6.963	4.000
Green Mountain	3.667	2.213	10.323	4.333
Highland Russet	3.000	0.857	6.517	4.667
Hindenburg	2.500	0.837	6.323	3.667
Ida Rose	4.000	5.483	9.637	2.667
Inca Gold	2.667	1.767	12.583	1.667
Irish Cobbler	3.250	3.393	8.170	3.000
Ivory Crisp	1.333	0.163	3.593	2.000
Jacqueline Lee	2.667	0.973	9.467	3.667
Kalkaska	1.667	0.297	5.600	1.667
Katahdin	3.333	5.103	5.920	3.333
Kennebec	2.500	1.150	4.007	4.000
Keuka Gold	2.167	0.743	4.480	2.667
King Harry (NY131)	2.167	1.337	5.613	2.000
Klamath Russet	2.667	0.617	7.823	4.667
La Chipper	2.833	2.107	5.790	2.667
Lamoka (NY139)	1.167	0.117	5.590	2.667
Langlade	2.500	0.687	5.060	2.667
Lehigh	2.667	0.890	5.007	2.667

Lelah (W2717-5)	1.000	0.250	4.663	2.667
Liberator	1.333	0.470	6.613	2.667
MaineChip	1.167	0.210	7.330	2.000
Marcy	1.167	0.177	3.313	3.333
MegaChip (W1201)	1.667	0.193	4.487	2.667
Michigan Purple	3.500	3.710	6.953	2.667
Missaukee (MSJ461-1)	1.500	0.263	5.360	1.667
MN 02419	2.167	0.583	15.583	2.000
MN 02467	3.000	1.530	3.843	5.000
MN 02586	1.667	0.207	8.753	1.333
MN 15620	2.500	1.213	9.493	4.333
MN 18747	2.833	3.727	5.990	4.333
MN 19298	3.833	4.017	9.097	2.000
MN 19350	2.833	1.690	6.827	3.333
MN 19470	1.833	0.917	6.447	2.667
MN 96072-4	2.667	0.827	9.957	3.333
MN 99380-1	1.833	0.480	7.403	2.333
Modoc	3.167	1.940	5.510	2.667
Monona	2.167	0.293	6.030	2.667
Monticello	1.583	0.337	4.720	2.333
Mountain Rose	2.667	2.237	6.667	3.333
MSG227-2	1.333	0.120	3.727	3.333
MSH228-6	1.167	0.237	6.993	2.333
MSI005-20Y	2.833	1.620	8.163	2.333
MSJ126-9Y	1.067	NA	3.987	1.994
MSJ147-1	1.000	0.150	8.507	3.333
MSK061-4	1.333	0.517	5.307	2.667
MSK409-1	1.000	0.133	5.267	2.667
MSL211-3	4.000	7.073	8.430	3.333
MSL268-D	1.500	0.200	6.050	2.667
MSL292-A	1.000	0.127	5.123	1.000
MSL512-6	1.333	0.233	7.400	3.003
MSL766-1	2.000	0.340	8.550	2.000
MSM051-3	1.333	0.200	5.253	2.667
MSM171-A	4.333	13.907	7.997	3.333
MSM182-1	2.667	2.227	4.913	2.333
MSM246-B	1.167	0.110	5.400	2.000
MSN105-1	3.167	1.757	9.173	2.333
MSN191-2Y	1.000	0.277	3.977	2.667

MSN215-2P	3.667	5.450	12.510	3.000
MSP239-1	1.833	0.700	5.590	2.000
MSQ070-1	1.167	0.073	5.260	2.000
MSQ176-5	2.333	0.177	5.003	2.000
MSQ279-1	1.667	0.290	4.340	2.000
MSR061-1	1.333	0.177	4.117	2.000
MSR160-2Y	1.333	0.240	8.127	2.000
MWTX 2609-2RU	3.167	1.957	5.593	5.000
ND1215-1 (NDSU 6)	2.167	0.460	5.603	3.333
ND2858-1 (NDSU 4)	2.167	1.070	13.413	3.333
ND8229-3 (NDSU 5)	1.500	0.327	5.427	5.000
ND8555-8R (NDSU 3)	3.167	2.607	9.240	3.333
ND860-2	1.500	0.137	5.563	2.000
Nicolet (W2133-1)	1.167	0.103	3.257	1.667
Norchip	2.333	0.230	8.073	2.667
NorDonna	4.000	1.620	6.320	2.333
Northstar	2.333	0.793	5.330	3.333
NorValley	1.333	0.510	4.370	2.333
Norwis (FL 657)	1.833	0.463	5.430	2.667
NY115	1.167	0.173	5.927	2.667
NY121	2.333	0.623	7.153	1.333
NY136	2.833	0.420	6.907	3.333
NY140	2.167	0.943	5.750	3.333
NY142	4.167	5.093	11.823	2.333
NYE48-2	1.000	0.167	3.697	3.333
Ontario	3.333	3.160	7.023	2.667
OR00068-11	2.500	1.013	9.103	2.667
PA99N82-4	2.967	1.134	6.492	3.503
Patagonia (NDSU 2)	3.667	2.203	7.567	4.333
Peter Wilcox	2.000	0.720	5.787	3.333
Pike	1.167	0.100	3.633	2.333
POR01PG1-6	NA	0.613	6.297	4.994
POR01PG16-1	1.833	1.173	7.507	5.000
POR01PG22-1	2.500	0.794	4.637	5.000
POR02PG37-2	2.667	1.170	7.903	2.667
Premier Russet	1.833	0.167	8.263	4.333
Prince Hairy (NY235-4)	2.667	1.757	6.700	2.000
Purple Majesty	1.667	0.287	6.290	3.333
Purple Peruvian	1.667	2.313	9.317	5.000

Ranger Russet	3.000	0.577	8.363	5.000
Reba	1.333	0.317	4.823	3.333
Red Maria (NY129)	3.167	1.780	7.243	2.333
Reeves Kingpin	3.167	3.233	6.520	5.000
Rideau	3.167	1.263	8.643	2.667
Rochdale Gold- Doree	3.000	1.460	7.487	2.000
Rosa	2.167	0.430	6.807	2.667
Russet Burbank	2.667	0.980	6.937	4.667
Russet Norkotah-S3	3.167	2.233	8.163	4.333
Russet Norkotah-S8	3.500	2.160	6.167	4.333
Salem	3.167	2.653	9.317	3.333
Satina	3.500	3.177	6.960	2.667
Sebago	3.000	4.703	6.027	2.667
Sierra Gold	2.667	0.517	6.420	3.333
Silverton Russet	2.500	0.797	5.013	5.000
Snowden	1.167	0.127	4.277	2.000
Superior	2.833	2.387	6.607	2.667
TrailBlazer Russet (ROND95249-Russ)	1.833	0.340	5.147	4.333
Tundra (W2310-3)	1.000	0.120	4.357	2.333
Umatilla Russet	2.500	2.497	6.087	5.000
V1102-1	3.667	2.373	10.907	4.333
V1115-3	2.667	1.117	5.257	4.333
Villetta Rose	3.167	1.300	5.230	2.667
W1151rus	3.333	4.800	7.500	5.000
W2253-5rus	3.500	2.907	8.383	4.333
W2309-7	1.000	0.153	4.087	3.333
W2683-2rus	2.333	0.653	7.763	5.000
W4013-1	1.167	0.137	4.877	2.333
Wallowa Russet	2.667	0.523	6.663	5.000
Waneta (NY138)	1.167	0.140	3.870	3.333
Western russet	3.000	1.030	6.427	4.333
White Pearl	1.000	0.101	3.158	2.500
Willamette	1.067	NA	4.107	NA
Winema	3.167	2.307	6.123	3.333
Yankee Chipper	2.000	0.287	5.457	3.667
Yukon Gem	1.833	0.603	5.227	3.333
Yukon Gold	3.333	1.210	7.190	3.000
Rio Grande Russet	3.000	1.373	7.333	5.000
Atlantic	1.333	0.143	4.660	2.000
