# Supplementary material for: Retrospective View of North American Potato (Solanum tuberosum L.) Breeding in the 20th and 21st Centuries
Source: G3 (Bethesda). 2013 Jun 1;3(6):1003–13. doi: 10.1534/g3.113.005595 (PMC3689798; doi:10.1534/g3.113.005595)
Supplement: Supporting Information [file supp_g3.113.005595_FigureS1.pdf]

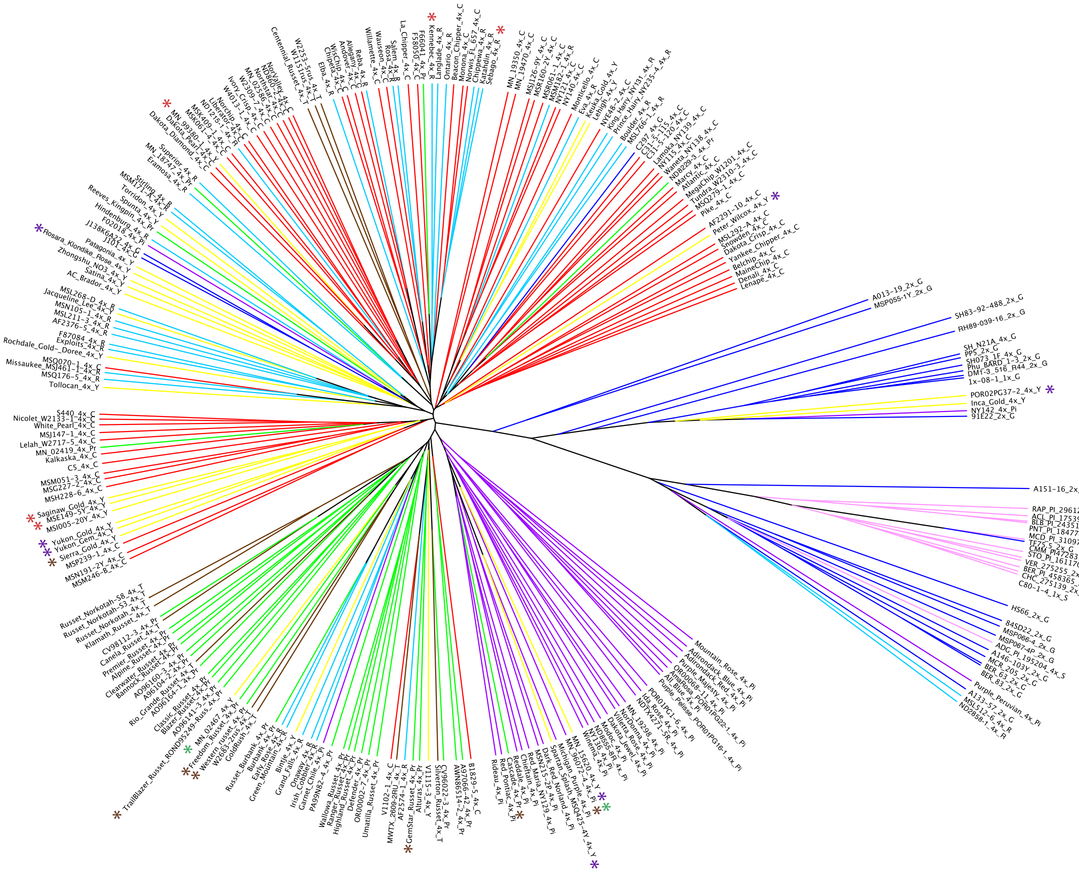

Figure S1 Unweighted pair group method with arithmetic mean (UPGMA) tree of 250 potato lines based on 3,763 single nucleotide polymorphism markers with dosage genotype calls and individual lines labeled. Color-coding is based on predominant market class designation and asterisks indicate lines that can be classified into more than one market class (red – Chip Processing; dark blue – Genetic Stock; purple – Pigmented; green - French Fry Processing; light blue – Round White Table; pink – Wild Species; brown – Table Russet; yellow – Yellow).
