# Supplementary material for: Retrospective View of North American Potato (Solanum tuberosum L.) Breeding in the 20th and 21st Centuries
Source: G3 (Bethesda). 2013 Jun 1;3(6):1003–13. doi: 10.1534/g3.113.005595 (PMC3689798; doi:10.1534/g3.113.005595)
Supplement: Supporting Information [file supp_g3.113.005595_TableS10.pdf]

Table S10 Summary of single nucleotide polymorphisms (SNPs) in carotenoid biosynthetic pathway genes tested for significant differences in allele or genotype composition compared to that observed in all other cultivated potato lines. SNPs with a p-value≤0.05 were considered significant.

PGSC Gene	Annotation	Significant Allele Frequency (Significant SNPs / Total SNPs)	Significant Genotype Frequency (Significant SNPs / Total SNPs)
PGSC0003DMG400013335	pTOX - alternative oxidase	0/1	0/1
PGSC0003DMG400028897	CHY1 - beta-carotene hydroxylase	1/1	1/1
PGSC0003DMG400008159	LCY-b - lycopene beta cyclase, chloroplastic	0/3	0/3
PGSC0003DMG400010637	LCY-b - lycopene beta cyclase, chloroplastic	0/2	1/2
PGSC0003DMG400009156	PDS - phytoene dehydrogenase, chloroplastic/chromoplastic	0/2	1/2
PGSC0003DMG400028224	CrtISO - carotenoid isomerase, chloroplastic	0/2	0/2
PGSC0003DMG400022473	ZDS - zeta-carotene desaturase	2/4	1/4
PGSC0003DMG400000333	LCY-e - lycopene epsilon cyclase	0/3	1/3
